# Is Police Misconduct Contagious? Non-trivial Null Findings from Dallas, Texas

**DOI:** 10.1007/s10940-021-09532-7

**Published:** 2022-01-12

**Authors:** Cohen R. Simpson, David S. Kirk

**Affiliations:** 1 Department of Methodology, London School of Economics and Political Science, London, UK; 2 Nuffield College, University of Oxford, Oxford, UK; 3 Department of Sociology, University of Oxford, Oxford, UK; 4 Leverhulme Centre for Demographic Science, University of Oxford, Oxford, UK

**Keywords:** Police, Misconduct, Social networks, Contagion, Survival analysis

## Abstract

**Objectives:**

Understanding if police malfeasance might be “contagious” is vital to identifying efficacious paths to police reform. Accordingly, we investigate whether an officer’s propensity to engage in misconduct is associated with her direct, routine interaction with colleagues who have themselves engaged in misbehavior in the past.

**Methods:**

Recognizing the importance of analyzing the actual social networks spanning a police force, we use data on collaborative responses to 1,165,136 “911” calls for service by 3475 Dallas Police Department (DPD) officers across 2013 and 2014 to construct daily networks of front-line interaction. And we relate these cooperative networks to *reported and formally sanctioned* misconduct on the part of the DPD officers during the same time period using repeated-events survival models.

**Results:**

Estimates indicate that the risk of a DPD officer engaging in misconduct is not associated with the disciplined misbehavior of her ad hoc, on-the-scene partners. Rather, a greater risk of misconduct is associated with past misbehavior, officer-specific proneness, the neighborhood context of patrol, and, in some cases, officer race, while departmental tenure is a mitigating factor.

**Conclusions:**

Our observational findings—based on data from one large police department in the United States—ultimately suggest that actor-based and ecological explanations of police deviance should not be summarily dismissed in favor of accounts emphasizing negative socialization, where our study design also raises the possibility that results are partly driven by unobserved trait-based variation in the situations that officers find themselves in. All in all, interventions focused on individual officers, including the termination of deviant police, may be fruitful for curtailing police misconduct—where early interventions focused on new offenders may be key to avoiding the escalation of deviance.

## Introduction

On May 25, 2020, Mr. George Floyd’s life was taken by then Minneapolis police officer Derek Chauvin. Three other police officers, two of whom were rookies, looked on as Officer Chauvin knelt on Mr. Floyd’s neck for a reported 9 min and 29 s. By the time Officer Chauvin removed his knee, Mr. Floyd had already taken his last breath.

*The New York Times* reported that Chauvin, now convicted of Mr. Floyd’s murder, had 22 official citizen complaints filed against him over the course of a 19-year career, many due to overly aggressive behavior (Barker and Kovaleski 2020). However, even though he had been reprimanded by the Minneapolis Police Department (MPD) for his misconduct, Chauvin had still been assigned duties as a training officer for new recruits, affording him the opportunity to shape the behavior of his colleagues.

Chauvin’s continued position of influence within the MPD despite his history of misconduct was not an isolated occurrence. In 2017, *The Washington Post* reported that nearly 1900 officers had been terminated from police departments in major metropolitan areas of the United States in the preceding decade because of improper behavior, with hundreds of these officers being reinstated after their removal was fought by police unions (Kelly et al. 2017). As the *Post* notes, “in many cases, the underlying misconduct was undisputed” and thus officers with a documented history of deviant behavior continued their employment—presumably working in close interaction with their colleagues. And, even amongst those officers who were not reinstated, some may have gone on to work for a different police department, where, for example, Grunwald and Rappaport (2020) find that 3% of police in Florida are so-called “wandering officers” who secure a job at another agency after being fired elsewhere.

The sustained presence of deviant officers in the halls of police departments stands to be hugely consequential to the integrity of law enforcement and, by extension, prospects for meaningful police reform and the development of trust between police and the communities they serve. This is because the social ties between members of a police force are important bases for occupational socialization, where sustained workplace relationships (e.g., friendship, line management, workgroup membership, instruction from a field training officer) and more ephemeral workplace interactions (e.g., assistance at the scene of an incident, advice sharing) present officers with numerous opportunities to learn what is and is not acceptable behavior (Chappell and Piquero 2004; Conti and Doreian 2010; Doreian and Conti 2017; Getty et al. 2016; Ingram et al. 2013, 2018; Lee et al. 2013; McNulty 1994; Ouellet et al. 2019; Paoline 2003; Quispe-Torreblanca and Stewart 2019; Skolnick and Fyfe 1993; Van Maanen 1974; Wood et al. 2019). Indeed, criminological scholarship on the adverse effects of police sub-culture—namely its ability to normalize malfeasance (Barker 1977; Chappell and Piquero 2004; Lee et al. 2013; Punch 2000, 2003, 2010)—suggests that the continued employment of officers with a history of deviant behavior may result in a scenario wherein the colleagues of these individuals (e.g., their partners, subordinates, and ad hoc collaborators) are themselves led astray over the course of regular interaction for the purposes of training, case work, and patrol. Consequently, here we investigate whether routine on-the-job interaction between members of a police force induces an interdependence of misbehavior, asking: if an officer is directly tied to others who step out of line, what impact, if any, might it have on her own propensity to misbehave? Put simply, might police misconduct be “contagious?”

To answer this question, we explicitly adopt a network perspective to examine the association between webs of collaborative workplace interactions and sanctioned misbehavior amongst uniformed officers of the Dallas Police Department (DPD) throughout 2013 and 2014. To dynamically map workplace collaboration networks, we rely on an accessible source of data that, to our knowledge, has never been used to formally measure police interaction—i.e., daily records of 911 calls for service. Like many police departments in the U.S., the DPD dispatches multiple patrol officers in separate vehicles to respond to 911 calls, particularly those deemed high priority. Such joint response to an incident creates a collaborative link between the officers involved as they work together to remedy the situation, where the scene of the incident itself presents an opportunity for discussion about acceptable behavior, the modelling of acceptable behavior, and general knowledge exchange (see McNulty 1994). Of particular interest here, however, is the agglomeration of joint responses to 911 calls, which, in aggregate, constitute a *dynamic*, *weighted *(*i.e., non-binary; valued*)*, department-spanning social network of front-line policing* wherein the association between any two officers is the number of times they collaborate over a given day.

Although we see considerable value in investigations of the role of organizational hierarchy (Quispe-Torreblanca and Stewart 2019; Ingram et al. 2013, 2018) and co-offending (Ouellet et al. 2019; Wood et al. 2019; Zhao and Papachristos 2020) in facilitating police misconduct, here we aim to contribute to the nascent body of research at the intersection of police deviance and social network analysis by using joint response to 911 calls to explore the behavioral implications of a broad set of direct, routine workplace interactions. Of course, whether police misconduct is found to be “contagious” likely depends on the nature of the intra-force social relationship that a researcher chooses to measure. Accordingly, some readers may balk at our decision to focus on daily, ad hoc workplace collaboration under the assumption that friends (i.e., “strong” social ties) and other informal acquaintances (e.g., confidantes, co-conspirators, and sources of advice) are more relevant conduits for social influence compared to the colleagues one is required to engage with at the scene of a 911 incident. Nevertheless, we maintain that the dense informal social networks that police officers build with one another from the point of recruitment (Conti and Doreian 2010; Doreian and Conti 2017) should be reinforced through formal on-the-job interaction (e.g., team membership and project collaboration), as in other organizations (e.g., see Ellwardt et al. 2012a; b; Lazega and Pattison 1999; Potter et al. 2015; Siciliano 2015).

## Social Networks and Police Deviance

Law enforcement officers stand to be powerfully drawn into errant behavior by their co-workers (Barker 1977; Punch 2000, 2003, 2010). This is especially so when considering the general segregation of officers from the public in addition to their need to repeatedly interact in close proximity and win the trust of their colleagues by conforming to occupational norms from the beginning of their careers (Merrington 2017; Savitz 1970; Van Maanen 1974; Waddington 1999). Indeed, police academies in the U.S. have been compared to medical schools as they function as “hot houses” that facilitate the formation of atypically dense social networks that can serve as vehicles for the transmission of occupational culture (Conti and Doreian 2010; Doreian and Conti 2017). In particular, academies enable recruits to gain a sense of what it means to be a “true” police officer with respect to: (1) formal policies and procedures; and (2) the informal, “common-sense” knowledge employed when dealing with the ambiguity inherent to law enforcement—where this occupational learning continues outside of classrooms, both “on the street” and off-duty (Conti and Doreian 2010; Doreian and Conti 2017; McNulty 1994; Moskos 2008; Paoline 2003; Van Maanen 1974).

Of course, acceptable conduct, as much as misbehavior, should be subject to peer effects (Chappell and Piquero 2004; Paoline 2003; Sutherland 1947). Acknowledging potential socialization into both positive and negative police behavior is essential due to the former being more common than the latter. Specifically, while there is considerable and persuasive quantitative evidence of racially-biased policing (see Knox et al. 2020; Ross et al. 2018), in general, serious forms of police deviance are expected to be rare relative to the sheer volume of police activity and police-public interaction typical of U.S. police departments (Worden 1996). Indeed, data presented in prior research, in addition to the data we analyze here, suggest that when bad behavior occurs it is likely to take the form of comparatively benign *police misconduct* such as administrative infractions, accepting free food, speeding unnecessarily, or sleeping on duty (see Chappell and Piquero 2004; Donner et al. 2016a; Huff et al. 2018; Kane and White 2009; Lersch and Mieczkowski 2000; Rozema and Schanzenbach 2019; Son and Rome 2004; Terrill and Ingram 2016; Quispe-Torreblanca and Stewart 2019). To be sure, grave on-the-job malpractice in the form of *police corruption* (e.g., modification of normal police behavior for reward; collaboration with criminals) and *police crime* (e.g., excessive and unjustified use of force; sexual assault) occurs—with dangerous and fatal consequences—but it is expected to be relatively unusual given the number of police and the volume of police activity.^1^ Consequently, officers should not be routinely exposed to colleagues engaged in serious forms of deviance (Rozema and Schanzenbach 2019; Terrill and Ingram 2016).^2^ And, by extension, *officers should encounter colleagues with varying histories of and attitudes about bad behavior in line with the patterning of social relationships throughout a police force*.

This network perspective on officers’ exposure to deviance via their co-workers (Ouellet et al. 2019; 2020; Quispe-Torreblanca and Stewart 2019; Wood et al. 2019; Zhao and Papachristos 2020) occupies a middle ground between the extremes of under- and over-socialized accounts of why police misbehave—i.e., “bad apples” versus “rotten barrels/orchards.” That is, a network perspective eschews a view of officers as “lone wolves” in order to focus on the behavior of police “in social relations” (Abbott 1997, p. 1152 and pp. 1165–1166; see also Brass et al. 1998). Simultaneously, it dispenses with the idea that officers are uniformly vulnerable to the influence of their aberrant colleagues by acknowledging that the social relationships between police, the behavioral implications of these relationships, and the behavior of police themselves are all far from monolithic. Consequently, a network perspective on police deviance has the great virtue of foregrounding intra-force heterogeneity such that it is consistent with conceptualizations of police sub-culture that emphasize how different officers will have distinct experiences with their colleagues, take diverging approaches to policing, and adhere to dissimilar, perhaps conflicting, behavioral logics around, for example, safety, competence, and machismo (see Campeau 2015; Herbert 1996; Herbert 1998; Ingram et al. 2018; Muir 1977; Paoline 2003; Paoline and Gau 2018; Son and Rome 2004).

Amongst scholarship focused on policing in the U.S., relational studies of deviance have generally been animated by social learning theory and, to a lesser degree, social control theory. The central premise of the former is that behavior is acquired through interaction with colleagues (e.g., field training officers and line managers) who model and normatively define action for some focal officer in a fashion that results in favorable or unfavorable views of deviance (Akers et al. 1979; Akers and Jennings 2015). On the other hand, social control theory posits that delinquent behavior is curtailed through officers’ positive socialization via strong ties to institutions and wider society (Wiatrowski et al. 1981).

In the various empirical applications of these two theories to the study of police, there is clearly the flavor of a network perspective on how officers encounter definitions of deviance (i.e., attitudes, values, and beliefs about inappropriate conduct) and, ultimately, come to misbehave (see, e.g., Chappell and Piquero 2004; Donner et al. 2016b; Getty et al. 2016; Lee et al. 2013; Wolfe and Piquero 2011). Indeed, some of this research comprises a promising, nascent empirical literature on the social networks of police (e.g., Ouellet et al. 2019; Roithmayr 2016; Wood et al. 2019). However, studies wherein criminologists actually analyze deviance alongside a social network spanning all, or some meaningful proportion of, a police department’s officers are rare. Moreover, in the few studies that explicitly relate the social networks of police to their deviant behavior, scholars have focused on less-traditional social ties by investigating either: (1) networks of indirect associations (cf. friendship) whereby officers are distally connected through the sharing of line managers (Quispe-Torreblanca and Stewart 2019); or (2) networks composed solely of deviant links between a subset of officers in a department who co-offend, thus excluding police with an untarnished disciplinary history (Ouellet et al. 2019; Wood et al. 2019; Zhao and Papachristos 2020). Because of these study designs, past relational research on police deviance within the traditions of social learning theory and social control theory cannot tell us whether an intra-force network of direct, non-deviant relationships might be associated with an officer’s propensity to engage in misconduct. This represents a notable gap in criminological understanding of police behavior vis-à-vis police sociality as direct, non-deviant relationships (e.g., friendship, advice provision, gossip, and collaborative exchange) are likely fundamental ties between a police department’s officers—where connections of this kind have previously been linked to deviance in other domains (e.g., see Gallupe et al. 2019; Paluck et al. 2016; Ragan et al. 2014).

Accordingly, we set out to quantitatively gauge the extent of the evidence in support of the idea that police misconduct is “contagious.” Specifically, we assess whether a propensity to engage in police misconduct is positively associated with patterns of *direct* and *routine* interaction (i.e., collaboration during the same calls for service) with other officers who have themselves engaged in misconduct in the past. In line with social learning theory, we expect that the risk of an officer engaging in police misconduct will increase when her direct exposure to deviant colleagues grows (Hypothesis 1). However, we acknowledge the possibility of positive (i.e., non-deviant) socialization as there is no theoretical basis for the presumption that only forms of deviant behavior are learned. Thus, we also expect that the risk of an officer engaging in police misconduct will decrease as her direct exposure to colleagues with untarnished disciplinary records grows (Hypothesis 2).^3^

## Methods

The primary data used for our analysis consist of: (1) the complete population of incidents generating 911 calls for service to the Dallas Police Department (DPD) *that DPD officers responded to* during 2013 and 2014 (i.e., 1,165,136 call-generating incidents, where 3475 officers responded to at least one incident); (2) official records of all *formally alleged misconduct that led to disciplinary action* against DPD officers between 2010 and 2014; and (3) demographic data on the employees of the City of Dallas (e.g., age, ethnicity, hiring date) between 2012 and 2017. Data on call response and disciplinary action were obtained through open records requests made directly to the DPD by the second author (Request Reference Numbers: 2015-06773 and 2016-04342). These requests specifically asked for information about all police officers responding to each respective 911 incident as opposed to just information about the first officer on the scene or the officer filing the subsequent incident report. Data on the employees of the City of Dallas were obtained from the City of Dallas’ online search tool for previously fulfilled open records requests (Request Reference Number: C000213-010818; https://dallascityhall.com/). See “Appendix 1” for details on how we link the three sets of data using officers’ badge numbers, names, and hiring dates as well as “Appendix 2” for a review of limitations of using procedurally generated data.

For our study, we focused on incidents deemed “high priority”—i.e., incidents classed by the DPD as an “emergency” (Priority 1) or simply as “urgent” (Priority 2)—*and* incidents deemed “low priority”—i.e., incidents classed by the DPD as “general service” (Priority 3) or “non-critical” (Priority 4). Approximately 52% of the incidents (603,544) are high priority.

All 3475 responding officers for 2013 and 2014 constitute the sample for our analysis. However, in constructing the daily collaboration networks spanning the DPD, we only draw a collaborative tie between these officers when they jointly respond to an incident that is: (1) small in size (i.e., five officers or less); and (2) circumscribed in duration (i.e., all officers assigned on the same day; short). These two restrictions are imposed to help bolster the integrity of our assumption that police at the scene of an incident directly interact (see “Appendix 3” for further explanation). Furthermore, these restrictions help protect our analysis from the potential impact of undercounting officers at the scene of large and protracted incidents. Specifically, official records of which officers are dispatched to which calls may not include ancillary officers who arrive on the scenes of incidents without informing the dispatcher. This is especially so for “hot” calls, such as those for officers in distress, during which there may be exceptional motivation for multiple officers to respond outside of the normal dispatch process in order to ensure officer safety—thus inflating the actual (undocumented) number of police associated with an incident and, possibly, its length.^4^ In total, 1,127,840 of the 1,165,136 incidents have five responding officers or less who all receive their assignment on the same day. These 1,127,840 incidents are used to construct the daily collaboration networks.^5^

### Dependent Variable

The outcome of interest for our study is a binary variable indicating, for each of the 730 days of 2013 and 2014, whether a given DPD officer in our sample engaged in any police misconduct *that was reported and ultimately met with disciplinary action*. To be clear, our dataset only includes information on alleged misconduct that was investigated and formally sanctioned by the DPD. The DPD did not provide us with information on any disciplinary cases wherein misconduct was alleged and did not result in disciplinary action of some form. Consequently, our dependent variable does not reflect unreported misbehavior or unsanctioned misconduct, instead only encapsulating the information we have on misconduct that resulted in some official form of disciplinary action, as formally documented by the DPD. Although our approach perhaps improves upon analyses of officers’ reports of their own misconduct (e.g., see Donner 2018, p. 6; Son and Rome 2004, p. 184), analyses of all instances of alleged misconduct regardless of outcome are the “gold standard” in police studies. Thus, a key limitation of our approach is that the DPD records we analyze may under-represent the frequency of deviance.^6^

Disciplinary action, in Dallas and elsewhere, can stem from a wide array of behaviors. Although infractions such as excessive use of force and the abuse of an individual in police custody are canonical examples of police deviance, acts such as the acceptance of free lunches and other small gifts have also been classed as errant by criminologists (Chappell and Piquero 2004; Punch 2000). Here we draw on work by Thomas Barker and David Carter, as cited in Donner et al. (2016a, p. 744), to view police deviance as activities that are inconsistent with officers’ legal and organizational authority and/or their standards of ethical behavior. This definition is broad enough to accommodate the myriad forms of deviance discussed in the criminological literature, namely: (1) job-specific malpractice that is nevertheless legal (Kane 2002); (2) the moderation of normal police behavior for some reward, and/or formal partnership with organized crime (Lauchs et al. 2011); and (3) the violation of legislatively-enacted laws (Donner et al. 2016a). We respectively class these forms of police deviance as *police misconduct*, *police corruption*, and *police crime*.

Returning to our data, we have information on the date that each disciplinary incident occurred, the date that an allegation of inappropriate behavior was received by the DPD, as well as the date that disciplinary action was taken by the DPD against the misbehaving officer. In total, there were 2651 disciplinary incidents involving one or more of the 3475 DPD officers in our sample between 2010 and 2014, inclusive. From these disciplinary incidents, we remove 15 wherein allegations of misbehavior were ultimately rescinded, or officers were ultimately exonerated, by department leadership. We also remove 131 “complex” disciplinary incidents wherein more than one of the 3475 DPD officers that responded to 911 calls in 2013 and 2014 were alleged to have been involved, analysis of which we eschew in order to ensure that we model the behavior of the individuals in our sample.^7^ This left us with 2506 disciplinary incidents involving 2703 instances of police deviance across the 3475 officers.

Most of the instances of deviance are forms of police misconduct and are relatively benign*.*^8^ Specifically, just 51 of the 2703 infractions are best classified as police crime (e.g., assault, unnecessary use of force, fraud, and sexual misconduct) whereas the remaining 2652 infractions are of an administrative nature and are best classified as police misconduct (see Supplementary Information [SI] Table 1 for our classification of the 134 unique infractions recorded by the DPD). Here we limit our attention to police misconduct as the small number of cases of police crime preclude large-scale quantitative assessment. Additionally, we restrict our analysis to the level of the day as this is the temporal granularity at which the disciplinary action records were constructed by the DPD. Consequently, we arrive at our dependent variable which, again, is a binary indicator for which of the 730 days of 2013 and 2014 that each DPD officer is recorded as having engaged in any police misconduct that led to disciplinary action (hereafter, “*sanctioned misconduct*”). We also stratify our regressions (discussed below) using the number of days that an officer is recorded as having engaged in any sanctioned misconduct prior to day $t$, counting from the beginning of our disciplinary action data in 2010.

Across the 3475 officers in our sample, 1082 of the 2,536,750 officer-day observations (3475 officers $\times$ 730 days) for 2013 and 2014 see an officer engage in one or more forms of sanctioned misconduct, where the number of previous “days of misconduct,” counting from 2010 until time $t$, ranges from 0 to 10 across the officer-day observations (see SI Table 2). For our analysis, we focus on the time until sanctioned misconduct (i.e., “day of misconduct” = 1 on some given day $t$)—a repeatable event—and whether this time is associated with an officer’s direct, on-the-job exposure to colleagues who themselves have engaged in sanctioned misconduct in the past and, conversely, colleagues who have not engaged in sanctioned misconduct in the past.^9^

### Independent Variables

Following Kane’s (2002) assessment of the spatial dependence of misconduct rates across police precincts, as well as prior studies of the network dependence of misconduct across police (Ouellet et al. 2019; Quispe-Torreblanca and Stewart 2019), our main correlates of interest are two “spatial lags.” In their most basic form, these spatial lags capture: (1) the weighted sum of a focal officer’s number of colleagues who engage in sanctioned misconduct on day $t$ (hereafter labelled “*calls with deviant colleagues*”); and (2) the weighted sum of a focal officer’s number of colleagues who *do not* engage in sanctioned misconduct on day $t$ (hereafter “*calls with non-deviant colleagues*”).

Formally, officer response to calls for service naturally constitutes two-mode (i.e., actor-by-event) networks which may be represented by a matrix with dimensions $N \times Z$ where, in the present case, $Z$ is the number of unique small, short call-generating incidents (whether high-priority or low-priority) occurring across a given day $t$ and $N$ is the number of DPD officers in our sample. As we are interested in any evidence suggestive of spillover in officers’ propensities to misbehave, we transform or “project” this two-mode structure to create a symmetric network represented by an $N \times N$ matrix $W$ that encodes the number of times any two DPD officers respond to the same 911 incident across a given day $t$. The connectivity matrix $W$ is then used to weight the behavior of those to whom an officer is directly connected, where the spatial lag for exposure to deviance is simply the sum of these weights. Specifically, this spatial lag is given by $\sum\nolimits_{j,i \ne j}^{N} {w_{ij}^{t} } y_{jt}$, where $N =$ 3475 officers, $w_{ij}$ is the cell in $W$ encoding the number of unique small, short incidents that officers $i$ and $j$ jointly respond to on day $t$, and $y_{t}$ is a binary vector indicating whether or not each of the $N$ officers engaged in any misconduct that was ultimately sanctioned on day $t$. To construct the spatial lag for non-deviant conduct, $y_{t}$ is simply inverted to $y_{t}^{\prime }$.

Valid estimates for the association between a spatial lag and some outcome of interest requires careful specification of $W$ as results stand to dramatically differ based on how this matrix is transformed. Importantly, $W$ should not be specified based on convention alone. Here we specify $W$ in a straightforward manner based on the directives of Neumayer and Plümper (2016). Specifically, we leave $W$ “as is”—i.e., a connectivity matrix of counts as described in the above paragraph. This results in the following assumptions. First, officers are differentially exposed to their peers (i.e., *heterogeneous* total exposure or, rather, all row-vectors in $W$ sum to different strengths/values). Second, the relative importance of the behavior of a colleague $j$ for some focal officer $i$ is fully determined by the number of collaborative events between $i$ and $j$ (i.e., tie strength). And third, those officers who share no collaborative events with $i$ on a given day $t$ are irrelevant to $i$’s behavior.

Although transformation of $W$ via row-standardization is typically done following influential work on “network autocorrelation” (e.g., Leenders 2002) and “spatial dependence” (e.g., Lacombe and LeSage 2018), Neumayer and Plümper (2016) forcefully argue against this practice, maintaining that row-standardization should be avoided without strong theoretical justification as it imposes an assumption of *homogenous* total exposure—i.e., all row-vectors in $W$ sum to unity—which erases between-actor (i.e., between-row) heterogeneity. While Neumayer and Plümper (2016) argue against row-normalization broadly, we avoid it specifically within the context of police studies as homogenous total exposure contravenes the aforementioned theoretical work on police sub-culture which, again, underscores the diversity of officers and their experiences (e.g., see Campeau 2015; Herbert 1996; Ingram et al. 2018; Paoline 2003; Paoline and Gau 2018).

Moreover, spatial lags of the form we adopt here clearly impose a strict assumption on how social influence is presumed to unfold—i.e., an officer’s behavior on a single day is only impacted by direct interaction with deviant/non-deviant colleagues across a single day. We can, of course, define much longer “exposure windows” of, for example, a few weeks or a few months. Yet, the existing criminological literature provides no theoretical justification for these choices and long exposure windows strike us as implausible due to the sheer number of incidents that typical patrol officers are generally required to respond to (Moskos 2007; Jaramillo 2019), such that cooperative experiences beyond the recent past may be quickly forgotten. On the other hand, a one-day exposure window is also arbitrary and, perhaps, overly restrictive.

Accordingly, we opted for a middle-ground approach by using the *cumulative sum* of calls with deviant/non-deviant colleagues, where this cumulative sum then “decays” each day to incorporate an element of “forgetting.” To clarify, consider, for example, a string of seven days starting on the first day of our observation period (i.e., January 1, 2013) for which a hypothetical officer has two “calls with deviant colleagues” on day one, one deviant call on day two, four deviant calls on day six, one deviant call on day seven, and no deviant calls on the remaining days—i.e., $\left\{ {2_{{t_{1} }} ,1_{{t_{2} }} ,0_{{t_{3} }} ,0_{{t_{4} }} ,0_{{t_{5} }} ,4_{{t_{6} }} ,1_{{t_{7} }} } \right\}$. Using a multiplicative factor whereby this officer’s cumulative sum for “calls with deviant colleagues” decays by 50% each day (i.e., 0.5), the set of values used to fit our models would be $\left\{ {2_{{t_{1} }} ,2_{{t_{2} }} ,1_{{t_{3} }} ,0.5_{{t_{4} }} ,0.25_{{t_{5} }} ,4.125_{{t_{6} }} ,3.0625_{{t_{7} }} } \right\}$. To calculate the first three numbers of the new set of values representing “calls with deviant colleagues” with a 50% decay: our hypothetical officer’s original value for “calls with deviant colleagues” at $t_{1}$ (i.e., 2) is taken as given; his original value for “calls with deviant colleagues” at $t_{2}$ (i.e., 1) is added to his original value at $t_{1}$ multiplied by the decay factor of 0.5 (i.e., $2 \times 0.5 + 1$) to get a new $t_{2}$ decayed value of 2; and his original value for “calls with deviant colleagues” at $t_{3}$ (i.e. 0) is added to his *decayed* value at $t_{2}$ (i.e., 2) multiplied by the decay factor of 0.5 (i.e., $2 \times 0.5 + 0$) to get a new $t_{3}$ decayed value of 1. The remaining values of “calls with deviant colleagues” with a 50% decay are then produced using the same arithmetic behind the decayed value at $t_{3}$ such that our hypothetical officer’s decayed value at $t_{4}$ is the result of adding his original value for “calls with deviant colleagues” at $t_{4}$ (i.e., 0) to his *decayed* value at $t_{3}$ (i.e., 1) multiplied by the decay factor of 0.5 (i.e., $1 \times 0.5 + 0$) to get 0.5, so on and so forth until the end of our observation period (i.e., December 31, 2014) and *mutatis mutandis* for “calls with non-deviant colleagues.”

Crucially, our approach balances: (1) concern that an officer is unable to remember every cooperative experience with their colleagues; with (2) an awareness that the behavioral consequences of an officer’s exposure to their colleagues on any given day may manifest over longer time scales. Furthermore, our approach sidesteps the “sharp transition” that is inherent to using the daily spatial lags by themselves (i.e., 100% decay) or taking the sum of calls with deviant/non-deviant colleagues using, for example, a rolling sum of three days, as these exposure-window-based approaches would see the value of the spatial lag drop instantly to zero at the end of each one-day/three-day period when an officer subsequently responds to no additional calls with deviant/non-deviant colleagues within the one-day/three-day window. In contrast, use of the daily cumulative sum with a daily decay allows the value of the spatial lag at time $t$ to continue to degrade (i.e., approach zero) each day until the end of our observation period or until an officer has more calls with deviant/non-deviant colleagues.

Of course, there is still an element of arbitrariness to our approach as the existing criminological literature does not indicate what decay factor one ought to use to best capture an officer’s “forgetting.” Thus, we opt for a small rate of decay—i.e., 5% or a decay factor of 0.95—under the assumption that an officer’s socialization will be both cumulative and salient. However, to judge the robustness of our findings, we also fit models using 10%, 20%, 30%, 40%, and 50% decay factors.

Last, note that the decayed cumulative sum of the daily spatial lags is itself temporally lagged by one day when fitting all of our models. That is, we use decayed cumulative calls with deviant/non-deviant colleagues on $t_{ - 1}$ to model sanctioned misconduct at $t_{1}$. We fit models in this manner to reflect our assumption that any network effect is unlikely to be instantaneous or, rather, that officers take time to react when exposed to the behavior of their ad hoc collaborators. Temporally lagging by one day also assuages concerns around reverse causality. This is because poor behavior may impact officers’ job assignments and thus their availability for call response and, by extension, front-line exposure to peers.

### Other Variables

We adjusted our models for the confounding influences of an officer’s gender, age, ethnicity, and department tenure. Furthermore, we adjusted for whether an officer was involved with patrol work on a given day $t$ (versus non-patrol tasks or not being at work) using a binary variable (*Non-Patrol Day*) that equals one for the days on which an officer responds to zero 911 calls for service of any severity, size, and length.

With census tract data from the 2012–2016 American Community Survey and geographic information for the analyzed calls for service, we also adjusted our models for the social context of policing. This is vital as prior research convincingly demonstrates that the nature of police work is contingent upon setting and situational characteristics, such that police are more likely to use force and to engage in misconduct in disadvantaged areas (Ba et al. 2021; Kane 2002; Kirk 2008; Parker et al. 2005; Reiss 1968; Sherman 1980; Smith 1986; Terrill and Reisig 2016). And our ability to account for key features of the social context of policing in a granular manner distinguishes our research from the few other networked-based studies of police misconduct and excessive use of force (Ouellet et al. 2019; Quispe-Torreblanca and Stewart 2019).

Specifically, for each of the 1,165,136 calls for service in our data, we matched its street address to the corresponding census tract to account for variation in the neighborhood conditions within which officers were tasked with responding to calls for service. This was done to create three binary indicators for whether, on a given day $t$, an officer responds to at least one 911 incident (of any severity, size, and length) in an area where: (1) at least 75% of the residents are Black (“predominantly-Black”); (2) at least 75% of the residents are Hispanic (any race) (“predominantly-Hispanic”); and (3) at least 40% of families are below the poverty line (“concentrated poverty”). For example, if an officer were to respond to four calls for service on day $t$, the binary indicator for exposure to areas of concentrated poverty would be equal to one if any of her four calls were in a census tract where the percentage of families below the poverty line is greater than or equal to 40% (i.e., a flag for whether a given officer spent some portion of her day responding to calls in an impoverished neighborhood). Accordingly, on those days wherein this officer responds to zero calls for service—or on those days wherein the calls she responds to have data for the poverty rate that are completely missing—her exposure to poverty for the purposes of responding to emergencies is assumed to be zero, *mutatis mutandis* for calls in predominantly-Black areas and calls in predominantly-Hispanic areas.^10^ Before model fitting, the binary indicators for predominantly-Black, predominantly-Hispanic, and concentrated poverty exposure are all temporally lagged by one day as, similarly to the spatial lags, poor behavior may impact officers’ job assignments and thus their availability for call response and, by extension, front-line exposure to deprivation and majority-minority areas.^11^

Descriptive statistics for all covariates appear in Table 1.

**Table 1:** Global descriptive statistics^a^

Variable	Description	Mean	SD	Min	Median	Max	Levels (% obs.)
Gender^b^	Female = 1; Male = 0	–	–	–	–	–	0 (84.4%)
							1 (15.6%)
Age	Years of age	40.34	9.79	20	40	70	–
Ethnicity^b^	1 = White						1 (52.8%)
	2 = Asian						2 (1.9%)
	3 = Black/African American	–	–	–	–	–	3 (25.4%)
	4 = Hispanic/Latino/Spanish						4 (18.7%)
	5 = Other						5 (1.2%)
Department Tenure	Number of days since being hired by the DPD divided by 365 and rounded to the nearest whole integer	13.34	9.62	0	11	47	–
Any Calls in Predominantly-Black Areas $(t_{ - 1} )$	1 = Officer responded to at least one 911 call for service on day $t$ located in a census tract wherein at least 75% of the population is Black; 0 = All calls on day $t$ in census tracts with populations less than 75% Black	–	–	0	–	1	0 (93.9%)
							1 (6.1%)
Any Calls in Predominantly-Hispanic Areas $(t_{ - 1} )$	1 = Officer responded to at least one 911 call for service on day $t$ located in a census tract wherein at least 75% of the population is Hispanic (any race); 0 = All calls on day $t$ in census tracts with populations less than 75% Hispanic	–	–	0	–	1	0 (93.8%)
							1 (6.2%)
Any Calls in Areas of Concentrated Poverty $(t_{ - 1} )$	1 = Officer responded to at least one 911 call for service on day $t$ located in a census tract wherein at least 40% of the families are below the poverty line; 0 = All calls on day $t$ in areas with less than 40% in poverty	–	–	0	–	1	0 (92.0%)
							1 (8.0%)
Decayed (5%) Cumulative Calls with Deviant Colleagues $(t_{ - 1} )$ ^c^	Cumulative sum with 5% daily decay of the number of 911 calls for service that an officer responds to on day $t$ with colleagues who engage in sanctioned misconduct at some point on day $t$	0.029	0.188	0	0	13	–
Calls with Deviant Colleagues $(t_{ - 1} \;{\text{to}}\;t_{ - 3} )$	The three-day rolling sum of the number of 911 calls for service that an officer responds to on day $t$ with colleagues who engage in sanctioned misconduct at some point on day $t$	0.004	0.099	0	0	13	–
Calls with Deviant Colleagues $(t_{ - 1} \;{\text{to}}\;t_{ - 7} )$	The seven-day rolling sum of the number of 911 calls for service that an officer responds to on day $t$ with colleagues who engage in sanctioned misconduct at some point on day $t$	0.010	0.151	0	0	13	–
Decayed (5%) Cumulative Calls with Non-Deviant Colleagues $(t_{ - 1} )$ ^d^	Cumulative sum with 5% daily decay of the number of 911 calls for service that an officer responds to on day $t$ with colleagues who *do not* engage in sanctioned misconduct at any point on day $t$	28.98	34.57	0	10.22	208.62	–
Calls with Non-Deviant Colleagues $(t_{ - 1} \;{\text{to}}\;t_{ - 3} )$	The three-day rolling sum of the number of 911 calls for service that an officer responds to on day $t$ with colleagues who *do not* engage in sanctioned misconduct at any point on day $t$	4.44	7.58	0	0	103	–
Calls with Non-Deviant Colleagues $(t_{ - 1} \;{\text{to}}\;t_{ - 7} )$	The seven-day rolling sum of the number of 911 calls for service that an officer responds to on day $t$ with colleagues who *do not* engage in sanctioned misconduct at any point on day $t$	10.35	14.15	0	2	123	–
Non-Patrol Day	1 = Officer not working on a given day or is not working in patrol (i.e., responds to zero 911 calls); 0 = Officer working patrol on day $t$	–	–	0	–	1	0 (26.7%)
							1 (73.3%)

^a^N = 2,232,677 officer-day observations (i.e., risk intervals) for 3278 Officers. Spatial lags only constructed using high-priority and low-priority calls with five responding officers or less who are all assigned on the same day

^b^Across the 3293 officers with available attribute data, the number in each racial/ethnic category is as follows: Asian (65), Black/African American (825), Hispanic/Latino/Spanish (613), White (1749), and Other (41). For these same 3293 officers, 507 are female and 2786 are male

^c^Descriptive statistics (i.e., Mean, SD, Min., Median, and Max.) for Decayed Cumulative Calls with Deviant Colleagues using decay factors greater than 5% (i.e., 0.95) are as follows: 10% (0.015, 0.133, 0, 0, 13), 20% (0.007, 0.096, 0, 0, 13), 30% (0.005, 0.08, 0, 0, 13), 40% (0.004, 0.071, 0, 0, 13), and 50% (0.003, 0.066, 0, 0, 13). Descriptive statistics for Decayed (5%) Cumulative Calls with Deviant Colleagues using: external/civilian-facing misconduct (0.004, 0.071, 0, 0, 12) and internal/department-facing misconduct (0.026, 0.176, 0, 0, 13)

^d^Descriptive statistics (i.e., Mean, SD, Min., Median, and Max.) for Decayed Cumulative Calls with Non-Deviant Colleagues using decay factors greater than 5% (i.e., 0.95) are as follows: 10% (14.68, 18.27, 0, 4.47, 128.9), 20% (7.38, 9.91, 0, 1.67, 103.29), 30% (4.93, 7.11, 0, 0.727, 96.61), 40% (3.70, 5.73, 0, 0.311, 92.95), and 50% (2.96, 4.91, 0, 0.125, 90.61). Descriptive statistics for Decayed (5%) Cumulative Calls with Non-Deviant Colleagues using: external/civilian-facing misconduct (29, 34.6, 0, 10.23, 208.62) and internal/department-facing misconduct (28.98, 34.58, 0, 10.23, 208.62)

### Modeling Strategy

To relate the spatial lags to sanctioned misconduct, we used a style of Cox regression for repeated events with complex dependencies and time-varying covariates that is broadly in line with the directives of Box-Steffensmeier and colleagues (Box-Steffensmeier and De Boef 2006; Box-Steffensmeier et al. 2007, 2014; Box-Steffensmeier and Jones 2009). Specifically, we made use of event-specific baseline hazards by stratifying our models by event number—here, the number of days that an officer has engaged in any sanctioned misconduct prior to day $t$, counting from the beginning of our disciplinary action data in 2010.^12^ Theoretically speaking, stratification reflects our assumption that an officer’s “days of misconduct” are dependent/conditional upon one another such that past misbehavior may shape how one acts in the future (Donner 2018). Practically speaking, stratification restricts the risk set (i.e., the set of officers at risk of engaging in misconduct on a given day $t$) such that the risk set for the $k$th “day of misconduct” is only comprised of the risk intervals (i.e., officer-day observations) for officers who have experienced $k - 1$ “days of misconduct”—where there are 2,536,750 *possible* risk intervals, or one risk interval for each of the 3475 officers for each day of 2013 and 2014 (i.e., $3475 \times 730$). Furthermore, our models include varying effects or “frailties” for each DPD officer in order to adjust for unknown, unmeasured, or unmeasurable factors that make some officers intrinsically more or less prone to engaging in misbehavior (i.e., actor-specific excess risk possibly attributable to factors such as a lack of self-control and/or lower inhibitions around rule-breaking that we cannot explicitly account for). As a result, the models used here are akin to multilevel models with random intercepts (Austin 2017).

Formally, and using the notation of Balan and colleagues (Balan 2018; Balan and Putter 2019, 2020), the model we estimated is as follows. Let $N_{i}$ represent an increasing, right-continuous counting process beginning on January 1, 2013 that is reflective of the event history of officer $i$, where $N_{i} \left( t \right)$ is the number of events (i.e., the number of “days of misconduct”) experienced by officer $i$ up to day $t$. Moreover, let $Y_{i} \left( t \right)$ represent an indicator function that equals one if officer $i$ is at risk of engaging in misconduct on day $t$ and zero otherwise. Here $N_{i}$ is modelled as a Poisson process and thus we are concerned with its *intensity*—i.e., the instantaneous probability of sanctioned misconduct on day $t$ given the entirety of officer $i$’s event history, or, rather, the force of transition from a “day of non-deviant behavior” to a “day of misconduct” given $N_{i} \left( t \right)$ (Andersen and Gill 1982; Balan 2018; Balan and Putter 2019, 2020). This may be contrasted with the familiar *hazard* for a single event which is the instantaneous probability of the event at time $t$ given that it has not yet occurred (e.g., death). Although “hazard” and “intensity” are sometimes used interchangeably (Prentice et al. 1981), here we use the latter terminology to highlight the counting process formulation of our models and to ensure consistency with Balan and Putter (Balan 2018; Balan and Putter 2019, 2020).

The intensity of the counting process $N_{i}$ for the $k$th strata (i.e., the $k$th “day of misconduct”) at time $t$, or $\lambda_{ik} \left( {t|Z_{i} } \right),$ is given as:

1 $$ \lambda_{ik} \left( {t|Z_{i} } \right) = Y_{i} \left( t \right)Z_{i} \exp \left( {\beta^{{\top }} \varvec{x}_{i} \left( t \right)} \right)\lambda_{0k} \left( t \right) $$

where $Z_{i}$ is the unobserved frailty (i.e., the random/varying effect) *shared across all risk intervals* for officer $i$ and $\lambda_{0k}$ is the unspecified, nonnegative baseline intensity for the $k$th strata/“day of misconduct” at time $t$ when all covariates are equal to zero. Furthermore, ${\varvec{x}}_{i} \left( t \right)$ is a $p \times 1$ vector of $p$ time varying and/or time invariant covariates for actor $i$ at time $t$, whereas $\beta$ is the corresponding $p \times 1$ vector of unknown parameters (i.e., the regression coefficients). Note that these parameters are conditional log intensity ratios which summarize the positive or negative association between some covariate of interest $x_{ip}$ and the intensity of the $k$th event for a one-unit increase in $x_{ip}$, where this association is multiplicative. It is assumed that event times are independent conditional on $Z_{i}$ and that the frailties themselves are independent and identically distributed in line with a distribution $Z$.

There is an active debate across the social and biomedical sciences around what distribution $Z$ should be assumed to govern frailties (Austin 2017; Balan 2018; Balan and Putter 2019, 2020; Box-Steffensmeier and Jones 2009; Hougaard 2000; Therneau et al. 2003). Here we fit our models using the gamma distribution for $Z$ in light of the simulation-based findings of Box-Steffensmeier et al. (2007, 2014) which indicate superior performance of stratified, repeated-events Cox models with gamma-distributed random effects in diverse scenarios typically of interest to social scientists—although the authors use gap (i.e., inter-event) time as opposed to the elapsed (i.e., calendar) time we employ here. In so doing, we rely on the “emfrail()” routine in Balan and Putter’s (2019) R package “frailtyEM” which: (1) adjusts standard errors for the parameter estimates $\hat{\beta }$ based on uncertainty stemming from the estimation of the gamma distribution’s scale parameter $\theta$; (2) provides a standard error and confidence interval for the estimated variance of the frailty terms; and (3) allows one to assess whether the proportional hazards/intensity assumption is met (i.e., the core assumption of Cox-style regression models) using the popular “cox.zph()” routine in Therneau’s (2018) R package “survival.” Assessment of whether a model specification inclusive of frailties is an improvement over that same model specification without frailties was done using a modified likelihood ratio test (see Balan 2018, pp. 37–40 and Balan and Putter 2019), which is the preferred formal assessment of $H_{Null} {:} \theta = 0$ (Box-Steffensmeier et al. 2007; Therneau et al. 2003).

Balan and Putter (Balan 2018; Balan and Putter 2019, 2020) provide additional formalism and a discussion of their expectation–maximization estimation procedure in relation to other implementations of frailty models popular in the social and biomedical sciences. Additionally, our appendices provide extended details on key decisions we took in relation to our modelling strategy. Therein we specifically discuss: (1) how we handle “time” with respect to the construction of the risk intervals and time-varying covariates (“Appendix 4”); (2) the exclusions we made to the set of 2,536,750 possible risk intervals in order to construct the final analytic risk set reflective of officers’ missing data and employment dates (“Appendix 5”); (3) how we go about stratifying our models (“Appendix 5”); (4) our rationale for the use of spatial lags and survival analysis over spatial regression (“Appendix 6”); and (5) our ability to identify “contagion” with observational data (“Appendix 7”).

## Results

**Fig. 1:**
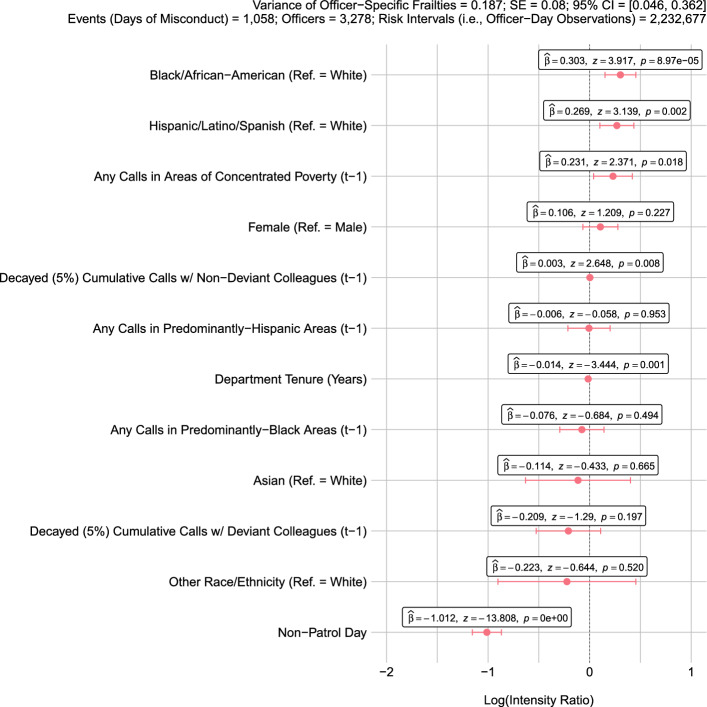
Covariate-specific risk of police misconduct (5% decay factor) for the Dallas Police Department (2013–2014). Parameter estimates $$\hat{\beta }$$ (log intensity ratios; bullets) in descending order and 95% confidence intervals from a repeated-events survival model of days until a Dallas police officer engages in sanctioned police misconduct alongside the estimated variance of the gamma-distributed frailty parameters capturing officer-specific excess risk (inset, above). Note, when reading *p* values, $$e$$ symbolizes base-10 scientific notation such that $$p = $$ 8.97e−05 $$ =$$ 8.97 $$\times 10^{ - 5} =$$ 0.0000897

Parameter estimates $\hat{\beta }$ (associational; non-causal) and their confidence intervals from our main model using a 5% decay factor are depicted in Fig. 1 in descending order by magnitude. With respect to interpretation, recall that $\hat{\beta }$ are estimated log intensity ratios (i.e., the bullet points in the figure). Accordingly, their exponentiation yields intensity ratios that summarize multiplicative shifts in the instantaneous probability of an event at time $t$—i.e., a transition from a day of non-deviant behavior to a day that includes one or more instances of sanctioned misconduct—*between two officers with the same frailty and with covariate vectors that are identical except for a difference of one unit in the covariate of interest*. Covariates with log intensity ratios greater than zero equate to additional “days of misconduct,” where the converse is true for log intensity ratios less than zero. With that in mind, estimates in Fig. 1 conflict with both of our hypotheses.

Regarding our first hypothesis, jointly responding to an additional call for service with a deviant colleague who has engaged in misconduct in the past is negatively associated with the intensity of a transition to a “day of misconduct.” Specifically, a one-unit increase in *Decayed (5%) Cumulative Calls with Deviant Colleagues* is associated with a reduction in the intensity of a transition to a “day of misconduct” by a factor of $\exp \left( { - 0.209} \right) = 0.811$ or 19%, holding the other covariates constant. One possible interpretation of this negative coefficient is that the sanctioned misconduct of a fellow officer deters other officers from engaging in similar behavior. However, the estimate is noisy and evidence against the null hypothesis of no effect is far from compelling (*p* value = 0.197).

In opposition to our second hypothesis, we find evidence to suggest that jointly responding to calls for service with colleagues who exhibited acceptable behavior in the past (*Decayed (5%) Cumulative Calls with Non-Deviant Colleagues*) is positively, rather than negatively, associated with the intensity of a transition to a “day of misconduct” ($\hat{\beta } = 0.003$; *p* value = 0.008), although the effect is miniscule. More specifically, an additional 911 call for service with a colleague who has not engaged in sanctioned misconduct is associated with an increase in the intensity of a transition to a “day of misconduct” by a factor of $\exp \left( {0.003} \right) = 1.003$, or 0.3%.

**Fig. 2:**
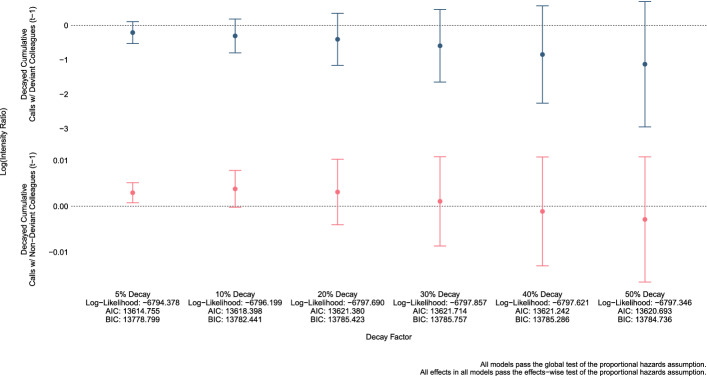
Risk of police misconduct for the Dallas Police Department (2013–2014) associated with *Calls with Deviant/Non-Deviant Colleagues* using decay factors from 5 to 50%, identical control variables, and identical risk intervals (see Fig. 1)

Turning to the sensitivity of our results, estimates $\hat{\beta }$ for *Decayed Cumulative Calls with Deviant Colleagues* and estimates $\hat{\beta }$ for *Decayed Cumulative Calls with Non-Deviant Colleagues* meaningfully vary when using different plausible decay factors. This is plainly demonstrated in Fig. 2 which plots $\hat{\beta }$ and their 95% Confidence Intervals for *Decayed Cumulative Calls with Deviant Colleagues* (top) and *Decayed Cumulative Calls with Non-Deviant Colleagues* (bottom) using the different decay factors while adjusting for the same variables seen in Fig. 1 and using the same 2,232,677 officer-day observations/risk intervals. In both instances, as the decay factor increases, the estimates become more negative and more uncertain; the latter of which is perhaps unsurprising given fewer officer-day observations with non-zero values under larger decay factors. Practically speaking, comparing the AIC and the BIC across the six models (Fig. 2; $x$-axis) indicates that the specification depicted in Fig. 1 using the 5% decay factor is (marginally) preferred. Nevertheless, it is clear that the value of the estimated coefficients associated with our two variables of interest are not robust to different plausible decay factors. Accordingly, the most conservative conclusion is that there is no association between *Decayed Cumulative Calls with Deviant Colleagues* and the intensity of misconduct nor between *Decayed Calls with Non-Deviant Colleagues* and the intensity of misconduct for our sample of DPD officers in 2013 and 2014. Thus, we fail to find support for either of our hypotheses.

Maintaining focus on the AIC-/BIC-favored model in Fig. 1 and turning to the other covariates, results echo prior research on the neighborhood context of policing (e.g., see Kane 2002) in that they suggest that the intensity of misconduct varies with the characteristics of the census tracts that officers patrol, specifically tract deprivation ($\hat{\beta } = 0.231$ for *Any Calls in Areas of Concentrated Poverty*; *p* value $= 0.018$). And, even when accounting for neighborhood context and involvement with patrol work ($\hat{\beta } = - 1.012$ for *Non-Patrol Day*; *p* value $< 0.005$),^13^ we also find compelling evidence that shifts in the intensity of misconduct are associated with the traits of individual officers. Specifically, and holding the other covariates constant, compared to White officers, the instantaneous probability of transitioning to a “day of misconduct” for Black officers and Hispanic/Latino/Spanish officers is estimated to be higher by a factor of $\exp \left( {0.303} \right) = 1.354$, or 35% (*p* value $< 0.005$), and $\exp \left( {0.269} \right) = 1.308$, or 31% ($p$ value $< 0.005$), respectively.

These findings are consistent with some prior research related to racial/ethnic differences in the likelihood of police misconduct as well as police violence (Kane and White 2009; Lersch and Mieczkowski 2000; Ridgeway 2016, 2020; White and Kane 2013). And because we adjust for the characteristics of the neighborhoods within which DPD officers respond to calls for service, we have attempted to estimate racial/ethnic differences in the intensity of misconduct net of any tendency for the DPD to systematically task its White, Black, Hispanic/Latino/Spanish, and Asian officers with call response in different areas of Dallas because of their race/ethnicity (Brown and Frank 2007). Nevertheless, our findings diverge from recent research that exploits fine-grained data on differences in officer duty assignments by race (Ba et al. 2021; Hoekstra and Sloan 2020), perhaps suggesting that the association between race/ethnicity that we observe would disappear with more comprehensive data. Moreover, because we are unable to adjust our models in a fashion that accounts for differential treatment of racial/ethnic groups by the DPD with respect to its disciplinary process, the coefficients could reflect a greater tendency for the misconduct of Black and Hispanic/Latino/Spanish officers to be recorded and sanctioned by the DPD compared to misconduct on the part of their White colleagues. Indeed, new research by Ralph (2020) documents at length the institutional racism and marginalization of non-White officers inside big city police departments. Hence, a “code of silence” may more commonly shield White police officers from being disciplined for their misconduct compared to Black or Hispanic/Latino/Spanish officers. We return to the race/ethnicity coefficients below vis-à-vis internal (i.e., department) and external (i.e., citizen) complaints.

As for the DPD officers’ other traits, department tenure is associated with a decrease in the intensity of misconduct by a factor of $\exp \left( { - 0.014} \right) = 0.986$ or 1% for each additional year on the job ($p$ value $< 0.005$). Substantively speaking, the intensity of misconduct would weaken, holding the other covariates constant, by a factor of $\exp \left( { - 0.014 \times 20} \right) = 0.756$, or 24%, if one were to move from being a relative newcomer with five years of experience to a veteran with twenty-five. Note that the parameter estimate for tenure maintains its negative expression when adjusting for officer age such that our findings likely reflect differences in conduct due to officer experience. However, age was dropped from the model specification in Fig. 1 due to its violation of the assumption that the intensities of officers being compared are constant through time (i.e., the proportional hazards assumption core to Cox-style regression).

Although the estimates summarizing covariate-specific shifts in the intensity of misconduct are interesting, a major strength of frailty models is the ability to go beyond observed heterogeneity in order to explore unobserved heterogeneity in the form of actor-specific excess risk that is unaccounted for by the set of covariates. In the present scenario, our model suggests that this risk merits discussion—although we stress that frailties could reflect unmeasured officer characteristics (e.g., personality) and/or unmeasured, misconduct-inducing situational factors that are simply correlated with unmeasured officer characteristics, especially given the recognized importance of situation to officer behavior (Ridgeway 2020).

**Fig. 3:**
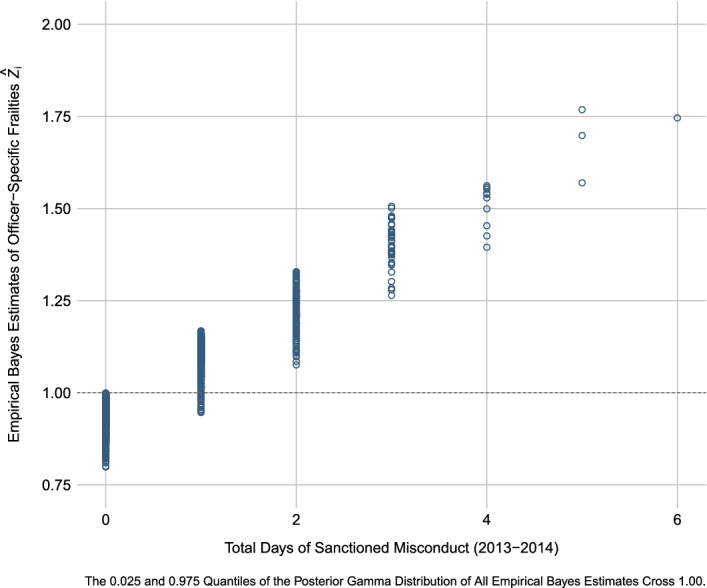
Officer-specific excess risk of police misconduct for the Dallas Police Department (2013–2014) based on the repeated-events survival model depicted in Fig. 1

Specifically, for the model depicted in Fig. 1, the *p* value for the likelihood ratio test comparing the fit of the model with frailties to the fit of the same model without frailties is 0.003, indicating the presence of excess risk; where the estimated variance of the gamma-distributed frailties for the DPD officers is approximately 0.187 (i.e., $1{/}\theta$; where $\hat{\theta } = 5.355$). To make sense of this heterogeneity, consider Fig. 3, which depicts the estimated frailties $\hat{Z}_{i}$ (i.e., empirical Bayes estimates; hollow bullets) from the model in Fig. 1 for the 3278 officers with valid risk intervals (see Table 1 and “Appendix 5”) in relation to these officers’ total number of “days of misconduct” experienced during the observation period. With respect to interpretation, a frailty indicates that were two officers to have *identical* observed covariate vectors and be observed over the same time period, the officer with the larger frailty would have a higher intensity of misconduct and thus more “days of misconduct,” where the frailty multiplicatively impacts the baseline intensity as in Eq. (1). For this particular sample of police officers, this impact is estimated to range, holding the covariates constant*,* from a roughly 20% reduction in the intensity of misconduct (i.e., zero total “days of misconduct” in Fig. 3) to a roughly 75% increase in the intensity of misconduct for those officers who repeatedly engaged in sanctioned misbehavior across the observation period (i.e., six total “days of misconduct” in Fig. 3). The positive relationship between the number of days that an officer engaged in misconduct that was ultimately sanctioned and their excess risk of future misconduct appears to be stark for our sample—a finding that is consistent with evidence recently presented by Donner (2018) suggesting a “state dependent” component of police behavior whereby prior misconduct shapes an officer’s propensity to misbehave in the future. Nevertheless, a strong association between the number of events and the frailties is to be expected (see Hougaard 2000, pp. 316–319) and the empirical Bayes estimates are all rather noisy, perhaps due to the few events per officer across the observation period (Mean = 0.323)—where the 0.025 and 0.975 quantiles of the Posterior Gamma Distribution of all estimated frailties $\hat{Z}_{i}$ cross 1.00.

Last, we assessed the sensitivity of our results to decisions around specification by estimating a series of additional models using permutations of the set of correlates in Fig. 1. Given limits on journal space, we present the figures depicting these ancillary models in the online-only Supplementary Information for our paper which is hosted alongside our data and code on the Open Science Framework: https://osf.io/9ypx6/. SI Fig. 1 and SI Figs. 2 through 6 respectively depict estimates from the aforementioned age-adjusted model and the aforementioned models using *Decayed Cumulative Calls with Deviant/Non-Deviant Colleagues* constructed with decay factors ranging from 10% to 50%. SI Fig. 7 depicts estimates from a model fit using *Decayed* (5%) *Cumulative Calls with Deviant/Non-Deviant Colleagues* and that adjusts for no social ecology indicators, which could moderate the impact of race/ethnicity on the intensity of misconduct as non-White officers may be disproportionately assigned to communities more conducive to misbehavior—namely those that are disadvantaged and that feature more crime (White and Kane 2013). SI Fig. 8 depicts estimates from a model fit using *Decayed* (5%) *Cumulative Calls with Deviant/Non-Deviant Colleagues*, the controls in Fig. 1, and only those risk intervals for the days on which officers respond to one or more 911 calls for service of any severity, size, and length (i.e., only those risk intervals where *Non-Patrol Day* = 0). SI Figs. 9 and 10 depict estimates from models that take an exposure-window-based approach whereby *Decayed (5%) Cumulative Calls with Deviant/Non-Deviant Colleagues* is replaced with the temporally lagged rolling sum of calls with deviant/non-deviant colleagues over the past three days (i.e., $t_{ - 1}$ to $t_{ - 3}$) and over the past seven days (i.e., $t_{ - 1}$ to $t_{ - 7}$). Finally, SI Figs. 11 and 12 depict results from models wherein we disaggregate our main outcome variable into binary indicators for external (i.e., civilian-facing) and internal (i.e., department-facing) misconduct.

Results using the various alternative model specifications are generally similar to those seen in Fig. 1, where none provide evidence in support of our two hypotheses. Note that in the models for which we disaggregate our dependent variable to distinguish between external and internal misconduct (SI Figs. 11 and 12), the association between race/ethnicity and the intensity of misconduct only persists for disciplined misconduct based on internal allegations (i.e., $\hat{\beta }_{{{\text{Black}}\;({\text{Internal}} \;{\text{Misconduct}})}} = 0.349$, $p$ $< 0.005$; $\hat{\beta }_{{{\text{Black}}\;({\text{External}}\;{\text{Misconduct}})}} = - 0.084$, $p$ $= 0.715$; $\hat{\beta }_{{{\text{Hispanic/Latino/Spanish}}\;({\text{Internal}}\;{\text{Misconduct}})}} = 0.320$, $p$ $< 0.005$; $\hat{\beta }_{{{\text{Hispanic/Latino/Spanish}}\;({\text{External}}\;{\text{Misconduct}})}} = - 0.149$, $p$ $= 0.574$). That is, it is only for internal forms of misconduct—the most common of which include behaviors such as violation of sick leave policy, violation of off-duty employment policy, and absence without official leave—and not external misconduct (e.g., threatening statements and illegal searches) that we observe an association with race/ethnicity. As stated above, findings may result from Black and Hispanic/Latino/Spanish officers being: (1) more prone to misconduct; (2) assigned to areas more conducive to misconduct; (3) less likely to have their minor forms of misconduct hidden by a “code of silence;” and/or (4) held to a higher standard of conduct compared to their White counterparts. Regardless, our ancillary models suggest that the race/ethnicity of the officers in our sample is not associated with misconduct stemming from civilian-facing allegations—although note that the number of officer-day observations that see external misconduct is tiny at 115 compared to the number of officer-day observations involving internal misconduct (i.e., 964).

Finally, regarding model diagnostics, the models presented in Fig. 1 and in SI Figs. 1–12 all have *p* values for the global test of the proportional intensity assumption (i.e., Schoenfeld residuals compared against the Kaplan–Meier transformation of time) that are greater than 0.05. Save *Age* in the model using all of our covariates (SI Fig. 1), *Non-Patrol Day* in the external-misconduct model (SI Fig. 11), and *Ethnicity: Asian* in the internal-misconduct model (SI Fig. 12), covariates in our models all have *p* values for the effect-specific tests of the proportional intensity assumption that are all greater than 0.05. Note that results and *p* values for the tests of the proportional intensity assumption are not reproduced in Fig. 1 and SI Figs. 1–12 and may instead be accessed via our entire “R” workspace uploaded to the Open Science Framework: https://osf.io/g93m7/.

## Discussion

From a policy standpoint, understanding if and how misconduct spreads between members of a police organization is vital for determining how to curtail police deviance and thus the most efficacious path to tangible police reform. Accordingly, here we have probed whether police misconduct might be “contagious,” such that a propensity to engage in deviant behavior increases through direct interaction with officers who have themselves engaged in misconduct in the past. In doing so, we have tested for the operation of a micro-level relational mechanism reflecting our supposition that police deviance is the result of differential exposure to errant colleagues in line with the dynamic structure of the department-spanning social networks within which members of a police force are routinely embedded. Critically, this mechanism is in agreement with cultural explanations of police behavior and the thrust of prior applications of social learning theory and social control theory to the study of police deviance—both of which underscore the diversity of officers’ dispositions, experiences, social ties, and social interactions, and thus their behavioral outcomes (e.g., see Campeau 2015; Herbert 1996; Ingram et al. 2018; Ouellet et al. 2019; Paoline 2003; Paoline and Gau 2018; Quispe-Torreblanca and Stewart 2019; Wood et al. 2019). Nevertheless, empirical support for this relational mechanism was found to be lacking. Specifically, results from our case study of sanctioned misbehavior and ad hoc workplace collaboration amongst 3475 uniformed members of the Dallas Police Department provided no evidence to compellingly suggest the “contagion” of police misconduct. Rather, the observed and unobserved traits of individual officers—i.e., their tenure, disciplinary history, individual proneness, and, in some cases, their race—appear to have the clearest association with whom ultimately steps out of line.

Do our results, then, provide support for “bad apples” theories of police behavior which emphasize correlates of misconduct such as specific personality traits (e.g., authoritarianism) and a lack of self-control (see, e.g., Donner 2018; Donner et al. 2016a; Worden 1996)? Again, our findings do point to several individual-level correlates of misconduct as well as unobserved officer-specific heterogeneity in misconduct, even after adjusting our models for factors related to the locations wherein officers respond to 911 calls. Nevertheless, the individual-level associations we observe do not necessarily indicate “bad apples” in the sense that our findings are wholly driven by officers who are psychologically or temperamentally predisposed to engaging in misconduct. This is because some portion of the observed associations between officers’ traits and misconduct may still be explained by unmeasured or unmeasurable factors (e.g., omitted variables related to situation or social context).

For instance, regarding racial/ethnic differences in officer behavior, Ba and colleagues (2021, p. 696) recently stressed that “rigorous evaluation of the effects of police diversity has been stymied by a lack of sufficiently fine-grained data on officer deployment and behavior that makes it difficult or impossible to ensure that officers being compared are facing common circumstances while on duty.” While we adjusted our models for the characteristics of the neighborhoods wherein officers responded to calls for service, our data are limited as we lack the kind of granular information on daily assignments and officer deployment that Ba et al. use in their study of the Chicago Police Department. Indeed, Ba and colleagues (2021, p. 699) were able to compare the behavior of Black versus White officers “given the same patrol assignment, in the same month, on the same day of the week, and at the same shift time.” Thus, in addition to the differential standards of conduct mentioned in the Results section, the associations between race/ethnicity and misconduct that we observe may in part stem from Black and Hispanic/Latino/Spanish officers being formally assigned to duties that tend to place them in situations that make misconduct more likely (e.g., patrol duty, in contrast to community policing or traffic duty) or assigned to certain shifts of work (e.g., night) or to days of work (e.g., weekends) that produce more situations conducive to misconduct.

Similarly, research suggests that less-experienced officers tend to be more active, patrol more aggressively, and initiate more citizen contacts than more experienced officers (see Worden 1996 for a discussion). Accordingly, that less-experienced officers in our data are associated with a greater intensity of misconduct may be a byproduct of these officers being in more situations conducive to misconduct rather than these officers being “bad apples” in the sense that they are inclined to disregard departmental policies. Moreover, as one of the lessons that officers learn in training and from their peers is how to “lay low” in order to avoid undue attention from superiors (see Paoline 2003), more experience yields a greater ability to evade situations conducive to misconduct as well as a greater understanding of how to conceal one’s misbehavior if desired.

This latter issue about concealing behavior underscores the limitations of relying exclusively on procedurally generated administrative data to study the behavior of police. Although our approach has allowed us to analyze official records of conduct for a substantial number of officers, we have zero control over data quality such that we may be missing unrecorded incidents of police misconduct—especially as our analyzed data only concern officially reported and formally sanctioned deviance. Of course, this lack of control is inherent to any study relying on documents and datasets created by third parties such as government agencies and private firms. Nevertheless, use of department records is particularly problematic to the extent that police sub-culture is characterized by codes of silence whereby deviance is under-reported (Cancino and Enriquez 2004; Ivković 2003). Furthermore, our use of department records is extra troublesome to the extent that misconduct is systematically overlooked for the purposes of compiling official records—particularly if, hypothetically speaking, the DPD had a culture of “noble” deviance (Punch 2000; Wolfe and Piquero 2011) during the study period whereby rules were routinely “bent” or widely flouted in the interest of, for example, “better” policing outcomes (e.g., an arrest) or bolstering officer safety (e.g., use of emergency lights, speeding, and brandishing weapons in association with putatively mundane incidents; see Sierra-Arévalo 2021).

Ultimately, what we can say given our analysis is that for the particular type of social tie examined in this study—i.e., collaborative interactions formed through joint responses to 911 calls—we do not find evidence suggestive of the “contagion” of police misconduct. In practical terms, what solutions should then be pursued to address police deviance? Keeping top of mind that our study is observational and based only on two years of data from just one large U.S. police department, results suggest that interventions focused on individual officers, including the termination of repeat offenders, may be fruitful for curtailing police malfeasance irrespective of the social connections that deviant officers have to other members of a police force. Indeed, recent work by Rozema and Schanzenbach (2019) focused on the behavior of thousands of police officers, detectives, and sergeants employed by the Chicago Police Department (CPD) indicates that the vast majority of these individuals were not problematic. That is, Rozema and Schanzenbach (2019) find that just 1% of the CPD employees (120 in total) received the bulk of citizen complaints and generated the costliest damage pay-outs in litigation—circa $6 million between 2009 and 2014 (not including legal fees)—leading the authors to also conclude that interventions should be concentrated on the very worst offenders. And, as our results suggest that a history of sanctioned misconduct fuels future misbehavior (see also Donner 2018), early interventions focused on new offenders may be key to avoiding the escalation of deviance (Ridgeway 2018) and the formation of the egregious offenders observed by Rozema and Schanzenbach (2019)—especially if the relatively minor forms of police deviance examined here are precursors to more dangerous behavior such as officer-involved shootings (Ridgeway 2016; see also Punch 2000, pp. 315–317). That said, the efficacy of removing problem officers is not without debate, as evidenced by a recent lively exchange between Chalfin and Kaplan (2021) and Sierra-Arévalo and Papachristos (2021). Specifically, whereas evidence originally presented by Chalfin and Kaplan (2021) suggests that incapacitating “bad apples” within a police department will have modest effects on deviance, Sierra‐Arévalo and Papachristos (2021) revisit Chalfin and Kaplan’s (2021) findings to instead demonstrate notable reductions in misconduct when removing problem officers should network spillover in police behavior occur.

To conclude, we return to the idea that whether police misconduct is found to be “contagious” may depend on the nature of the measured relationship between the officers of interest. Thus, we end by raising the possibility that “contagion” may indeed play out across the DPD, but that the formal on-the-job interactions we have studied here are simply not conduits for social influence despite our opening assertion that they should reinforce and encourage the informal intra-force social ties which, based on prior research (e.g., see Gallupe et al. 2019; Paluck et al. 2016; Ragan et al. 2014), are likely implicated in workplace social learning (e.g., friendship, advice giving). Two considerations lead us to consider such a conclusion.

First, recall that collaborative partners for the purposes of responding to 911 calls for service are, in many respects, forced upon each DPD officer such that these individuals are likely to have little to no control over who they get to work with. As discussed in detail in “Appendix 7,” homophily (i.e., choice of social contacts based on similarity stemming from factors such as race, taste, and personality) seriously confounds studies of peer influence (Shalizi and Thomas 2011). Yet, the workplace social contacts that officers actively choose may be far more relevant to their behavior. And, as Doreian and Conti’s (Doreian and Conti 2017; Conti and Doreian 2010) ethnographic research on the formation of a friendship-like network inside a U.S. police academy demonstrates, members of law enforcement may clearly prefer social relationships with some colleagues over others, despite the overall number of connections between members of a force being large (i.e., high network density).

Second, both Ouellet et al. (2019) and Quispe-Torreblanca and Stewart (2019) find that officers exposed to colleagues who have engaged in malfeasance are indeed more likely to engage in improper behavior. Recall that the manner in which social ties are drawn between police in both of these studies differs starkly from our own. Again, we have given primacy to direct, ad hoc collaborative interactions for the purposes of 911 call response which make up an important component of routine law enforcement. On the other hand, Ouellet et al. (2019; see also Wood et al. 2019; Zhao and Papachristos 2020) analyze co-offending relationships (i.e., whether Chicago police officers are co-named in a formal complaint), while Quispe-Torreblanca and Stewart (2019) focus on connections reflective of formal organizational hierarchy (i.e., whether London police officers are in the same “peer group” simply defined as being “assigned to the same line manager”). Although both pioneering studies are clever in their designs, they respectively rely on: (1) deviant relations amongst a subset of deviant officers who are perhaps predisposed to stepping out of line, excluding relations these individuals have to *non-*deviant peers; and (2) institutional connections that may or may not reflect meaningful direct interaction. Indeed, in designing our analysis, we have heeded the recommendations of the authors of these pioneering studies to examine the broad social structure of police departments by analyzing officers’ behavior in relation to ties to both deviant and non-deviant colleagues (Ouellet et al. 2019, p. 18; Wood et al. 2019, p. 14).^14^

Still, the differences in our approach and that of Ouellet et al. (2019) and Quispe-Torreblanca and Stewart (2019) raise the possibility that our conflicting findings are attributable to differences in how officers’ social relationships are measured as well as our analysis of deviant officers alongside non-deviant officers. And our research is not meant to be an exact or conceptual replication of the studies of Quispe-Torreblanca and Stewart (2019) and Ouellet et al. (2019) due to the substantial differences between the three studies with respect to relational data, dependent variable, length of time, setting, and methodology—where future research should aim to replicate and build upon all three studies.

All in all, there are multiple ways to define and operationalize the social relationships between members of a police force that are plausibly germane to: (1) building a greater understanding of how officers might influence one another; and, more broadly, (2) clarifying the behavioral implications of police sub-culture. However, future network research on police deviance ought to make a special effort to assess whether the theoretically and empirically consistent micro-level relational mechanism we have advanced here sees support when emulating Doreian and Conti (Doreian and Conti 2017; Conti and Doreian 2010) and Ouellet et al. (2020) by analyzing data on informal, voluntary intra-force social ties such as “friendship,” who officers prefer to “hang out with” off-duty, and who officers explicitly “look to for advice and guidance” (McNulty 1994; Paluck et al. 2016). Of course, the administrative data used by Ouellet et al. (2019), Quispe-Torreblanca and Stewart (2019), and ourselves represent easy ways to measure social networks that span an entire police force and thus model the behavior of officers at scale. Yet, a shift in focus to informal face-to-face social relationships measured in the most rigorous manner (see Lee and Butts 2018) using standardized survey prompts will enable future studies on the interplay between intra-force networks and police deviance to be more readily compared and synthesized in meta-analyses (e.g., see Gallupe et al. 2019 on adolescent friendship and deviance) in order to move towards definitive and actionable conclusions about police malfeasance.^15^ Furthermore, a focus on intra-force networks composed of officers’ informal, voluntary relationships will allow researchers to capitalize on models developed by sociologists for the coevolution of networks and behavior (see Greenan 2015; Gallupe et al. 2019; Snijders 2017)—arguably the “gold standard” for observational studies of peer influence via face-to-face networks—and, when appropriate, employ techniques to assess causality (see Aral and Nicolaides 2017; Eckles and Bakshy 2021; Shalizi and McFowland III 2018; and “Appendix 7”).

## Data Availability

All of our data, the “R” code used to transform and analyze our data, and a copy of the complete “R” workspace containing objects for the fitted models and figures are accessible via the Open Science Framework (OSF): https://osf.io/g93m7/. Additionally, the OSF project page for our paper, not the journal website, hosts the Online-Only Supplementary Information (https://osf.io/9ypx6/), which includes the four SI Tables and the twelve SI Figures referenced throughout this document.

## Appendix 1: Data Linkage

Using text fields containing the badge numbers of the 3475 officers who responded to at least one of the 1,165,136 call-generating incidents and simple Boolean matching, we linked our data on call response to our data on disciplinary action to relate collaboration to police deviance. Furthermore, we used text fields containing officers’ names to link our data on call response to basic information on officers’ characteristics obtained from a list of all individuals employed by the City of Dallas between 2012 and 2017.

For this second linkage, we started with a mixture of Boolean matching using officers’ first name and last name or their first name, last name, and middle initial in those cases where there are multiple individuals with the same first name and last name. Boolean matching was then followed with manual matching whereby the names of officers from the call data that were unmatched to employee records were visually compared to those in the employee data to catch keying errors such as misspellings and character inversions (e.g., Jeffrey versus Jeffery). During the manual matching, we assumed that if the first initial, middle initial, and last name of an officer from the call data corresponds to a singular first initial, middle initial, and last name entry in the employee records then the two individuals are the same. For example, “Jones, Jim J” is matched to “Jones, J.” We did not manually match names based solely on assumptions around marriage (e.g., two women who just have the same first name and second name/initial but different last names would not automatically be matched as the same person). Basic text pre-processing of names was done prior to the Boolean matching (i.e., conversion of text to lowercase and the removal of empty space, full stops, hyphens, and generational signifiers such as “Sr.” and “III”). Only the City of Dallas employee records with the City-provided descriptor “Dallas Police Dept—Uniform” were considered to be eligible for the Boolean and manual matching.

Last, to adjudicate in those rare situations where there were multiple officers with the same first name/initial, middle name/initial, and last name, the call data and employee records were supplemented with official records of response to resistance (i.e., use of force) between 2013 and 2016 which include officers’ badge numbers, ethnicity and dates of hire. With this additional information, we were able to manually link the name associated with a badge number in the call data to the name associated with a plausible employee record through the badge numbers and hiring dates in the use of force data and the hiring dates in the employee records. Exploitation of the use of force data in this manner was also used to adjudicate when officers have the same first name/initial and second name/initial but different last names in an attempt to catch officers who change their surname over time (e.g., due to marriage). All in all, we were able to match 3293 of the 3475 officers appearing in the 911 call data to names in the employee records. Data on response to resistance were obtained from the City of Dallas’ open data portal (https://www.dallasopendata.com).

## Appendix 2: Data Limitations

Although not atypical when examining employees of entire government departments, a key shortcoming of our analysis is the limited data we have about individual officers. Specifically, we only have information on an officer’s gender, ethnicity, birth year, date of hire, rank (e.g., Police Officer versus Police Senior Corporal), rate of pay, level of education, and employment status as of 2018. As we combined employee records from 2017 with call data from 2013 to 2014, we do not use information on rank and pay as both should vary from year to year across the DPD officers in a fashion that we cannot anticipate, making backdating such information highly suspect. As a result, gender, ethnicity, years of age, and the years since an officer was hired (i.e., department tenure/on-the-job experience) are the only covariates for the characteristics of the DPD officers available for our analysis.^16^

We do not use the information on education included in the previously fulfilled open records requests for the employee data (Request Reference Number: C000213-010818; https://dallascityhall.com/) as the spreadsheet detailing employees’ level of education was filled with seemingly numerous inaccuracies (e.g., missing data and multiple entries for the same individual), raising concerns about data quality and the potential undermining of our analysis through measurement error. As mentioned in the discussion section of the main text, this of course underscores the pitfalls of relying exclusively on procedurally generated administrative data.

## Appendix 3: Ensuring Models Reflect Meaningful Officer Engagement at the Scene

We restrict the set of 911 incidents used to construct the daily collaboration networks of the 3475 officers in our sample in two important ways. First, we do not draw a collaborative tie between officers when they jointly respond to an incident for which the number of officers on the scene is so large they cannot reasonably be expected to meaningfully engage one another (e.g., through information exchange, assistance with operational tasks, and/or emotional support). Such “large” incidents concern routine policing behavior in the form of, for example, backing up a fellow officer, responding to major disturbances, responding to major accidents, and dealing with burglaries, robberies, kidnappings/abductions, fires, shootings, stabbings, and animal attacks. Across the 1,165,136 call-generating incidents, the number of officers responding to each incident ranges from 1 to 98 (Median = 2). Here we favor a conservative cut-off at five responding officers which is the 97^th^ percentile for our data (99th Percentile = 8). Of the 1,165,136 call-generating incidents, 1,132,386 have five responding officers or less.^17^ Note that we measure the number of responding officers by simply counting the unique officer badge numbers associated with each high-priority and low-priority incident in the records provided by the DPD.

Second, we do not draw a collaborative tie between officers when they jointly respond to an incident unfolding over a substantial period of time. Like those incidents with very many responding officers at the scene, the “long” incidents in our data concern routine policing behavior, where it also seems unreasonable to expect that all officers assigned to an incident across multiple days will substantively interact with one another. To measure the number of days over which an incident unfolds relative to officer involvement, we simply use the elapsed time between the first and last dates on which responding officers are recorded by the DPD as being assigned to the incident. Across the 1,165,136 call-generating incidents, the number of days between the assignment of the first and the last responding officer ranges from 0 (all officers assigned at some point on the same day) to 32. As the vast majority of incidents see same-day officer assignments (1,159,143 out of 1,165,136), we exclude from our analysis those incidents unfolding over multiple days.^18^

In total, 1,127,840 incidents have five responding officers or less who are all assigned on the same day. These incidents are used to construct the daily collaboration networks.

## Appendix 4: Survival Analysis and the Handling of “Time”

To accommodate our time-varying covariates, we followed the recommendations of Box-Steffensmeier and Jones (2009) and Therneau et al. (2018) to use the “counting process” formulation of the Cox model (Andersen and Gill 1982) whereby risk intervals are constructed for each officer for each day of our observation period—i.e., 3475 $$\times$$ 730 or 2,536,750 possible risk intervals. These risk intervals or “officer-day observations” are open on the left and closed on the right with the general form ($$t_{ - 1}$$,$$ t$$], where $$t \in \left\{ {1, \ldots ,730} \right\}$$ and the time-varying covariates corresponding to $$t$$ are treated as constant over the daily intervals.

Note that we diverge from Box-Steffensmeier and colleagues (Box-Steffensmeier and De Boef 2006; Box-Steffensmeier et al. 2007, 2014; Box-Steffensmeier and Jones 2009) and use “elapsed/calendar time” since the beginning of our observation period (i.e., the start of our 911 call data on January 1, 2013) as opposed to inter-event or “gap time.” Under the latter temporal scheme, counting would begin anew with the interval ($$0$$,$$ 1$$] on the day after any sanctioned misconduct occurs such that the titular gaps would be the number of days between “days of misconduct.” As Box-Steffensmeier and Jones (2009) discuss, the gap-time formulation of a repeated-events Cox model is the appropriate choice when the data generating process is such that the risks for events develop *sequentially*. In contrast, elapsed time reflects the assumption that risks can develop *simultaneously*. Here we strongly prefer elapsed time as we jointly analyze diverse infractions under the umbrella of “police misconduct” (see SI Table 1) and the assumption that an officer would, for example, only consider sleeping on duty *after* she improperly discharged her weapon strikes us as implausible even if the realization of the former and the negative consequences it entails “carry over” to impact the risk of the latter (hence, our stratification by event number/“days of misconduct”).

## Appendix 5: Construction of the Risk Set and Model Stratification

Although there are 2,536,750 possible risk intervals, not all are valid for the purposes of our analysis. The most obvious constraint on the risk set is that there are intervals of time for which some of the 3475 officers are not at risk of engaging in police misconduct because they had not yet been hired by the start of our observation period in 2013 or because they had left the department before the end of our observation period in 2014. Moreover, it is not inconceivable that some officers step away from 911 call-response duties due to sick leave, vacation, suspension, and job rotation (e.g., a period of time doing desk work). Furthermore, some unknown fraction of the 3475 officers may have non-patrol core assignments and thus appear in the 911 call data not because responding to calls for service is their main responsibility, but rather because they are responding to select calls in a supervisory capacity or for some other idiosyncratic reason.

These occupational scenarios point to complex issues around one’s availability for call response and when exactly they might be at risk of engaging in police misconduct. As opposed to attempting to devise bespoke strategies to handle each scenario using the limited data we have about the individual DPD officers,^19^ we choose to instead handle them all with an uncomplicated exclusion rule and a straightforward assumption. Specifically, with respect to employment transition, we excluded from our analysis those risk intervals for the days prior to an officer being hired and those risk intervals for the days after an officer’s employment ends using the hiring and termination dates in the aforementioned employee records from the City of Dallas.^20^ Additionally, we assume that the risk intervals for the days on which an officer does not respond to high-priority calls for service or low-priority calls for service are valid—i.e., officers who are not involved in call response of any kind on a given day $t$ can (and indeed do) engage in misconduct.

Critically, this assumption allows us to model the behavior of officers who have not been fired but who do move into and out of call response during our observation period whilst avoiding arbitrary assumptions about availability and the risk of misconduct. For example, we could have removed those risk intervals for which the number of days since an officer responds to calls for service is greater than zero or some other short time period (e.g., seven days, 30 days). Similarly, we could have removed those risk intervals for the days before an officer that we know is employed first appears in our data on calls for service (i.e., before they take their first call in 2013 or 2014). However, these exclusion rules would require us to make the odd assumption that misconduct by an officer who responds to calls on one day and misconduct by that same officer on a day wherein he responds to no calls are somehow distinct behaviors that should be modelled separately. That said, officers who are simply not engaged in call response on a given day, who move away from call response over the observation period (for whatever non-fireable reason), or who rarely appear in the call data may systematically differ in their inclination towards misconduct compared to colleagues actively engaged in call response. Accordingly, we include in our models a binary indicator for whether a risk interval is for a “non-patrol day”—i.e., *Non-patrol Day* equals one for those risk intervals wherein an officer responds to zero high-priority *and* zero low-priority calls of any size and length—which, along with tenure, should adequately capture variation in officers’ propensities to engage in misconduct vis-à-vis their career stage.

Last, recall that there are 1082 “days of misconduct” (i.e., 1082 risk intervals wherein officers engage in sanctioned misbehavior) across the 3475 responding officers in the call data but that we were only able to match 3293 of these officers to the City of Dallas employee records. As the R-based implementation of the Cox-style model that we use does not accommodate incomplete observations, we excluded from our analysis the risk intervals for the 182 officers without information for their basic characteristics using listwise deletion. Of these excluded risk intervals, 15 see officers engage in one or more forms of police misconduct that is ultimately sanctioned. Moreover, as we temporally lag some of our covariates that vary over time by one day and construct exposure-window-based spatial lags for the past three days and the past seven days, officers are given missing values for some correlates for up to seven of their initial risk intervals from January 1, 2013. These risk intervals, nine of which see officers engage in sanctioned misconduct, are also removed with listwise deletion.

After the exclusions related to officers’ missing data and their employment dates, we are left with 2,232,677 risk intervals for 3278 officers which we used to fit all of our models, save the ancillary model using the subset of officer-day observations for which *Non-patrol Day* equals zero (SI Fig. 10). Across the 2,232,677 risk intervals, there are 1058 “days of misconduct,” where the number of previous “days of misconduct” up to time $t$ and counting from January 1, 2010 ranges from 0 to 10.^21^

This results in 11 possible strata based on officers’ known event histories, where officers who have never been sanctioned for misconduct by the DPD on or after January 1, 2010 based on the information we have are included in the first stratum (i.e., at risk of their first “day of misconduct”). The number of risk intervals that see officers engage in misconduct ranges from 455 to 0 across the 11 strata. As a stratified Cox model is akin to fitting separate models for each stratum, the small numbers of events in the upper strata stand to impair estimation. Accordingly, we “collapse” or top-code the number of strata for our models in order to stabilize the number of events in each stratum in line with the recommendations of Box-Steffensmeier et al. (2007, p. 246).^22^ A qualitative assessment of event counts by stratum indicates that using four strata (i.e., one, two, three, and four or more “days of misconduct”) is most sensible as this results in at least 100 “days of misconduct” across the officers per stratum. Note that officers move into a new stratum on the day immediately following a “day of misconduct.” We also top-coded the number of strata to fit the ancillary models focused exclusively on external and internal misconduct (SI Figs. 11 and 12, respectively; see also SI Table 1).

See SI Table 2 for the number of risk intervals that contain sanctioned misconduct in each stratum and SI Table 3 for the manner in which we recoded officers’ race and ethnicity. Those unfamiliar with counting process formulation and risk set construction should see SI Table 4 for a schematic of how we arranged our data. Descriptive statistics for the 2,232,677 risk intervals appear in the main text in Table 1.

## Appendix 6: Rationale for Survival Analysis

Readers versed in spatial regression may wonder why we did not use a spatial model for a limited dependent variable, specifically, the Time Series Cross-Section (TSCS) variant of the well-known spatial probit model (Lacombe and LeSage 2018; Franzese et al. 2016). We rely on Cox-style regression and spatial lags instead of a TSCS spatial probit model for the following reasons. First, the sheer amount of data we use for our analysis make the simulation-based strategies required to appropriately estimate the latter model computationally infeasible [see both Calabrese and Elkink (2014) and Franzese et al. (2016) for discussions]. Given $$N =$$ 3475 officers and $$T = $$ 730 days of study, the TSCS spatial probit model would need to be fit to the $$N \times T =$$ 2,536,750 officer-day vector indicating which days officers engaged in sanctioned misconduct whilst using an $$NT \times NT$$ block-diagonal connectivity matrix wherein a series of $$N \times N$$ submatrices along the prime diagonal would encode daily cooperation amongst the DPD officers (i.e., $$W^{t}$$). Of course, we could have aggregated our data to the level of the week, month, or even the year to ease computational burden. However, we feel that aggregation unnecessarily wastes information on the timing of behavior as the data we used to construct our dependent variable are granular, with the DPD recording misbehavior at the level of the day. Furthermore, use of a spatial probit model would raise vexing issues around: (1) the ordering of misconduct and calls for service within larger time windows; and (2) when an officer should be included in the model given the occupational constraints mentioned in our discussion of the construction of the risk set above.^23^ On this latter point, to the best of our knowledge at the time of writing, no spatial probit model for dynamic data whereby actors enter and exit a network/connectivity matrix has been devised. Last, misconduct itself is quite infrequent. And although a promising “rare events” spatial probit model has been proposed (Calabrese and Elkink 2016), simulations by the model’s architects suggest a high false positive rate relative to other estimators. All in all, given the constraints of our data, we feel that the strengths of Cox-style regression outweigh any weakness relative to our aims—although we underscore that the assumption that frailties, baseline intensities, and event times are independent is far from ideal given our explicit focus on network dependence.^24^

Finally, note that there are multiple flavors of survival analysis for recurrent events that may give different results (Balan and Putter 2020; Box-Steffensmeier et al. 2007, 2014; Kelly and Lim 2000). Thus, a brief theoretical justification of our preference for a shared-frailty Cox model stratified by event number is warranted. Past criminological research indicates that assuming both event dependence and actor heterogeneity is prudent. Specifically, the former reflects the “slippery slope” perspective on misconduct implicit in some maligned sub-culture arguments that position low-level misbehavior (e.g., acceptance of free food) as a gateway to more serious forms of deviance (see Punch 2000, pp. 315–317; and Dean et al. 2010). Although this view of police deviance as a progressive process is not without criticism, principally around the idea that moral decline proceeds as an irreversible downward spiral (see Merrington 2017, pp. 54–56), we maintain that it is plausible that past misconduct alters an officer’s propensity for future bad behavior whilst not necessarily ensuring that deviance will come to pass [see also Donner (2018) on “state dependency” and reoffending amongst police]. Moreover, as we mention in the main text, an assumption of officer-specific heterogeneity in propensities to misbehave is appropriate given our lack of information on the attributes of officers likely to be implicated in their decision to step out of line (e.g., their personality traits and situational factors correlated with their job assignments). Furthermore, an assumption of officer heterogeneity is consistent with studies on police sub-culture that emphasize the diversity of officers and their experiences (e.g., Campeau 2015; Herbert 1996; Ingram et al. 2018; Paoline 2003; Paoline and Gau 2018).

## Appendix 7: A Note on Identifying “Contagion” with Observational Data

We would be remiss if we did not provide a cautionary note regarding our ability to identify contagion using an observational research design. Network data are powerful and present an excellent opportunity to explicitly investigate the behavioral implications of “social structure.” However, the limitations associated with measuring the influence of network peers on the behavior of individual network members using observational research designs should not be understated.

One of the most important contributions to understanding of this issue is a landmark paper by Shalizi and Thomas (2011) who persuasively argue that homophily or “social selection” (i.e., network formation due to sharing traits such as gender or race) and social influence (i.e., “contagion” or “peer effects”) can never be distinguished from one another in observational studies. The authors’ conclusion stems from the idea that homophily due to some latent (i.e., an unmeasured or unmeasurable) trait, as opposed to manifest homophily in accordance with some observed trait (e.g., gender or ethnicity), will *always* preclude the identifiability of social influence. This is expected to hold even if one has managed to measure and adequately account for a large number of factors through which social selection might operate.

Nevertheless, recent work does provide some defense of the utility of observational data for answering research questions related to social influence. For example, Eckles and Bakshy (2021) promisingly detail how one might use propensity score models to indeed adjust for a very large number of factors—in their case thousands—to increase the accuracy of estimates of peer effects. Moreover, Shalizi and McFowland III (2018) argue that if a given network forms according to latent homophilic tie formation then its structure should fully encode such tendencies. Accordingly, if one adjusts their model by incorporating information on actors’ positions within the latent social space summarizing the network of interest (either two-dimensional Euclidean space or through latent non-overlapping clusters) then, theoretically*,* one should be able to retrieve unbiased estimates of peer effects. Additionally, Aral and Nicolaides (2017) rely on instrumental variables estimation to assess the contagion of running behavior, exploiting exogenous variation stemming from the varying weather experienced by geographically distant friends to derive unbiased estimates of peer effects.

Unfortunately, however, we are unable to make use of these strategies for two reasons. First, we have very limited background information about the DPD officers (see “Appendix 2”)—where the information we do have does not present us with a naturally occurring source of variation in officers’ rates of misconduct that is plausibly uncorrelated with the behavior of their ad hoc collaborators (cf. Quispe-Torreblanca and Stewart 2019 who skirt this weakness through clever use of information on misconduct and officer line management). Second, the large number of officers in our sample, in addition to the complexities around their entry into and exit from the daily collaboration networks (see “Appendix 5”), make an appropriate method of estimating latent space models and stochastic block models in the manner laid out by Shalizi and McFowland III (2018) unclear.

That said, the risk of confounding due to homophily, both latent and manifest, is arguably reduced in our study due to the manner in which DPD officers in different vehicles ultimately come to respond to the same 911 calls. To be clear, it is erroneous to say that front-line collaboration, as we have measured it, is “random”—especially given the range of unobserved factors that surely govern call response (e.g., the micro-dynamics of officer availability). However, to the extent that there are DPD officers who wish to control their emergency-response-related assignments, their limited ability to select which 911 calls they respond to should work against homophily. This is especially so when comparing joint-response to 911 calls to other voluntary social relationships that researchers have linked to individual behavior such as, for example, who officers might choose to conspire with (Ouellet et al. 2019; Wood et al. 2019; Zhao and Papachristos 2020), who they choose as friends (Conti and Doreian 2010; Doreian and Conti 2017; Gallupe et al. 2019; Ragan et al. 2014), or who they explicitly look to for advice and guidance—i.e., their “social referents” (McNulty 1994; Ouellet et al. 2020; Paluck et al. 2016).

In the past, there was relatively strict adherence to “beat responsibility” amongst DPD officers whereby they would generally avoid responding to 911 calls beyond their beat’s boundaries. However, the massive glut of 911 calls that presently characterizes emergency support in Dallas (Jaramillo 2019) has resulted in a situation where dispatchers typically direct the closest available officers to the scene of a call. Moreover, the DPD, like law enforcement agencies in other major metropolitan areas of the U.S., often struggles to keep up with call volume (Jaramillo 2019) such that there is little time left over for patrol officers to engage in discretionary, self-initiated activity. Moskos (2007, p. 147) refers to the futile attempt to respond to all calls, often at a frantic pace, as the “tyranny of dispatch,” and we expect it to strongly work against social selection.

Indeed, a recent study and staffing analysis conducted by KPMG (2019) found that Dallas patrol officers engage in self-initiated proactive work only about 15% of the time, meaning that the bulk of their time is spent responding to 911 calls (see also Weisburd et al. 2015). This is worth noting because measuring collaboration between DPD patrol officers using their response to 911 calls does in fact allow us to capture their primary source of on-the-job interaction; where the decision on which officers will be present at the scene of 911 incidents is generally taken by the dispatcher.

All things considered, these characteristics of call response—in addition to our need to carry out a longitudinal analysis of relatively rare behavior at a high temporal resolution across a large, dynamic set of individuals—led us to pursue a straightforward methodological strategy in the form of Cox-style regression with random effects and spatial lags (see also “Appendix 6”). As a result, our analysis comes with an important caveat—i.e., *our models only allow us to assess whether a propensity to engage in misconduct might be correlated with patterns of DPD officers’ direct workplace interactions with colleagues who have engaged in sanctioned misconduct in the past. We can make no causal claims and our results should be viewed as correlational*.

We must also underscore that we can draw no conclusions as to whether our findings, in whole or in part, stem from the presence or absence of the broad institutional norms and organizational features consistent with the notions of “systemic corruption,” “systemic misconduct” and “rotten trees/orchards” (see Sherman 1978; Skolnick and Fyfe 1993; Wilson 1968). To empirically and defensibly draw affirmative or negative conclusions about systemic deviance, whether observationally or causally, would require fundamentally different data—ideally a mixture of both qualitative and quantitative (Ross et al. 2018) from very many police departments for a proper population-level analysis (Alpert and MacDonald 2001; Chappel et al. 2006; Ivković 2003, 2009; Terrill and Ingram 2016)—and a different modelling strategy. As a result, our express intention here is to narrowly gauge the amount of correlational evidence in favor of a micro-level mechanism of police misconduct whereby focal officers misbehave in the wake of exposure to deviant behavior through routine intra-force interaction. This mechanism is more limited in scope, more analytically tractable, and more consistent with the available evidence on rates of serious police deviance compared to the broad-based, negative socialization posited in scholarship concerned with the pernicious effects of entire policing institutions. Somewhat similarly, our data and analysis cannot speak to any systemic and/or disproportionate use of aggressive and repressive forms of policing (e.g., as in the case of stop-and-frisk, protest policing, use-of-force, no-knock raids, etc.) against specific subsets of the U.S. population (e.g., Black Americans and the poor) and its adverse effects.

## Footnotes

1 That said, past work may underestimate various forms of police deviance due to, for instance, “codes of silence.” Furthermore, certain police behaviors may be uncommon and still disproportionately and adversely impact subsets of the U.S. population. For example, Ang (2021) observes that police killings in Los Angeles are rare but are nevertheless consequential to the educational attainment of witnessing Black and Hispanic students (see also Legewie and Fagan 2019; Ross et al. 2018). Moreover, uncommon police behaviors could still adversely impact the perceived legitimacy of police, which has important implications for productive public-police interaction (see Jackson et al. 2021).

2 We, of course, recognize that defining the “seriousness” of various forms of police deviance is a subjective matter. In no way do we mean to discount the deleterious consequences of fairly common police behavior such as stop-and-frisks, verbal threats, and intimidation. Rather, our intent is to point out that there is a variety of different types of police deviance, and, thankfully, the available evidence suggests that the majority of the 700,000 officers working in the approximately 13,200 local police departments in the United States (FBI 2020) are unlikely to engage in grave acts such as murder, sexual assault, and corruption.

3 Note that our goal is not to test hypotheses of “perfect imitation,” “behavioral mimicry,” or “direct transmission” (e.g., the diffusion of smoking cigarettes, use of a handgun, or infection with COVID-19). Instead, we aim to investigate whether an officer’s propensity to misbehave in some fashion is associated with the observed, past misconduct of colleagues. Put alternatively, our concern is whether sanctioned misconduct on some day $t$ by some focal officer is partly a function of working previously with colleagues who have a demonstrated disposition for rule-breaking (or rule-following), where our analysis is premised on the assumption that this disposition stands to “rub off” on the focal officer. Although our approach is more general than studies of imitation/mimicry by scholars of social networks (e.g., see Greenan 2015), it is wholly consistent with past work on police deviance vis-à-vis intra-force relationships (e.g., see Quispe-Torreblanca and Stewart 2019) as well as with criminological theorizing around differential association and social learning. Indeed, in their review of the intellectual foundations and condition of empirical research around social learning theory, Akers and Jennings (2015) state that “…personal definitions favorable or unfavorable to crime and deviance can be expressed as general definitions (e.g., covering a wide range of behaviors) or specific to a particular behavior or to a particular situation” (Akers and Jennings 2015, p. 233). Thus, the theory of Akers and colleagues (Akers et al. 1979; Akers and Jennings 2015) does not mandate analyses of police peer effects that only concern imitation/mimicry, although such analyses are of course within the remit of social learning theory. In this respect, our study is best understood as a broad test of social learning.

4 We are very grateful to Reviewer #4 for suggesting that we further consider the nature of response to “hot” calls.

5 We acknowledge that our analysis may still undercount officers at the scene of each incident if the “hotness” of calls in Dallas is uncoupled from incident size and incident length as documented in the 911 call data. Unfortunately, without additional information, the impact of “hot calls” cannot be completely addressed.

6 The formal actions taken by the DPD against an employee in a disciplinary incident (which may feature multiple forms of deviance) include documentation of the incident in the officer’s employee file in addition to written reprimands, counselling, training, suspension, and termination. Incidents may also be overturned upon appeal to relevant authorities. The outcomes of disciplinary incidents, however, are not the focus of this research.

7 There is one “complex” incident wherein the allegation against one of two officers in our sample was rescinded. This incident is analyzed as an instance of misconduct only for the second officer for which the allegation was upheld and punishment given.

8 This does not mean that police misconduct is of no concern. Importantly, it implies inadequate control and supervision of officers, incompetence, and the avoidance of work—all of which stand to facilitate more serious misbehavior (Donner 2018; Punch 2000; Ridgeway 2016). As a result, police misconduct is worthy of attention.

9 We favor using an aggregate measure of misconduct for our main analysis rather than disaggregating allegations of misconduct derived from internal sources (e.g., allegations made by fellow officers or supervisors) versus external sources (i.e., allegations resulting from a citizen complaint). The data provided to us by the DPD details the outcomes of the disciplinary process, not the source of the initial complaint. Accordingly, there is some subjectivity around definitively classifying allegations as external (i.e., civilian-facing) or internal (i.e., department-facing) using the limited text-based descriptions employed by the DPD. Furthermore, disaggregating the disciplinary data stands to impair the quality of our models (stratified Cox regression for repeated events; discussed below) due to the small number of instances of sanctioned police misconduct, particularly external misconduct (see Box-Steffensmeier et al. 2007, p. 246). Nevertheless, we do assess the sensitivity of our main model when disaggregating our outcome variable by internal and external sources. To classify a given form of misconduct as internal or external, we relied upon prior work (e.g., Wood et al. 2019) as well as information from the DPD Internal Affairs Division (2020). See SI Table 1 for our classification of the DPD’s 134 unique infraction codes as external or internal.

10 Out of the 1,165,136 911 incidents, 40,948 are located at addresses that we could not match to a census tract of Dallas. And across the 2,536,750 officer-day observations, there are 5682, 5682, and 6065 wherein an officer responds to calls for which data on the percentage of Black residents, the percentage of Hispanic residents, and the percentage of families below the poverty line are all missing, respectively.

11 Note that we lack complete, longitudinal information on officer’s job assignment and shifts of work. Although the data on call response provided by the DPD does indicate which unit of the force each responding officer belongs to as well as the beat of Dallas that an incident occurs in, extrapolation of unit and beat assignments across the officer-day observations of 2013 and 2014—which number over two million—would be problematic as we would not know if or when officers switch units and beats outside of the context of the call data itself. Thus, we would have to assume that the call data fully encode unit and beat assignment, which we do not think is advisable—especially when officers only appear in the call data intermittently.

12 We could also count from the beginning of the call data on January 1, 2013. Both points in time are of course arbitrary. However, counting from 2010 allows us to incorporate additional information on officers’ event histories.

13 The striking protective association between misconduct intensity and not being involved in patrol work is perhaps due to officers with non-patrol duties: (1) being in fewer situations that increase the risk of misconduct (e.g., desk work); (2) being better at their jobs and thus eligible for more favorable assignments involving less interaction with citizens in situations characterized by uncertainty; and/or (3) occupying supervisory roles—where these individuals were presumably promoted because they adhere to departmental rules and are less inclined to engage in misconduct.

14 Although Quispe-Torreblanca and Stewart (2019) are one of the few network studies to analyze ties between deviant and non-deviant officers, Ingram et al. (2018) approach the issue of socialization in a similar manner by examining officers who belong to the same workgroup (also see Ingram et al., 2013; Lee et al. 2013; Getty et al. 2016).

15 For instance, even if police union contracts constrain the ability of police executives to terminate problematic officers, executives will still have the power to: (1) isolate problematic officers by assigning them to duties wherein their ability to facilitate deviance may be neutralized (i.e., intelligent dispatch and deployment); and (2) disband work relationships found to be combustible (i.e., actively “design” or “engineer” intra-force social networks through “tie removal”).
